# Genome-wide identification of novel expression signatures reveal distinct patterns and prevalence of binding motifs for p53, nuclear factor-κB and other signal transcription factors in head and neck squamous cell carcinoma

**DOI:** 10.1186/gb-2007-8-5-r78

**Published:** 2007-05-11

**Authors:** Bin Yan, Xinping Yang, Tin-Lap Lee, Jay Friedman, Jun Tang, Carter Van Waes, Zhong Chen

**Affiliations:** 1 Head and Neck Surgery Branch, National Institute on Deafness and Other Communication Disorders, National Institutes of Health, Center Drive, Bethesda, Maryland 20892, USA; 2 Laboratory of Clinical Genomics, National Institute of Child Health and Human Development, National Institutes of Health, Convent Drive, Bethesda, MD 20892, USA; 3 Department of Preventive Medicine, University of Tennessee, Health Science Center, N Pauline St., Memphis, TN 38163, USA

## Abstract

**Background:**

Differentially expressed gene profiles have previously been observed among pathologically defined cancers by microarray technologies, including head and neck squamous cell carcinomas (HNSCCs). However, the molecular expression signatures and transcriptional regulatory controls that underlie the heterogeneity in HNSCCs are not well defined.

**Results:**

Genome-wide cDNA microarray profiling of ten HNSCC cell lines revealed novel gene expression signatures that distinguished cancer cell subsets associated with p53 status. Three major clusters of over-expressed genes (A to C) were defined through hierarchical clustering, Gene Ontology, and statistical modeling. The promoters of genes in these clusters exhibited different patterns and prevalence of transcription factor binding sites for p53, nuclear factor-κB (NF-κB), activator protein (AP)-1, signal transducer and activator of transcription (STAT)3 and early growth response (EGR)1, as compared with the frequency in vertebrate promoters. Cluster A genes involved in chromatin structure and function exhibited enrichment for p53 and decreased AP-1 binding sites, whereas clusters B and C, containing cytokine and antiapoptotic genes, exhibited a significant increase in prevalence of NF-κB binding sites. An increase in STAT3 and EGR1 binding sites was distributed among the over-expressed clusters. Novel regulatory modules containing p53 or NF-κB concomitant with other transcription factor binding motifs were identified, and experimental data supported the predicted transcriptional regulation and binding activity.

**Conclusion:**

The transcription factors p53, NF-κB, and AP-1 may be important determinants of the heterogeneous pattern of gene expression, whereas STAT3 and EGR1 may broadly enhance gene expression in HNSCCs. Defining these novel gene signatures and regulatory mechanisms will be important for establishing new molecular classifications and subtyping, which in turn will promote development of targeted therapeutics for HNSCC.

## Abstract 2

Microarray profiling of ten head and neck cancer lines revealed novel p53 and NF-κB transcriptional gene expression signatures which distinguished tumor cell subsets in association with their p53 status.

## Background

Numerous basic and clinical studies suggest that development and malignant progression of cancer is rarely due to a defect in a single gene or pathway. Multiple genetic alterations accumulate during carcinogenesis, potentially leading to aberrant activation or suppression of multiple pathways and downstream genes that have important functions in determining the malignant phenotypes of cancer. Microarray technology has enabled us to study global gene expression profiles of cancers and identify gene programs or 'signatures' that are critical to the heterogeneous characteristics and malignant phenotypes of cancers, even of the same pathologic type [1-3]. In head and neck squamous cell carcinomas (HNSCCs), gene expression profiling has been used in attempts to identify biomarkers for diagnosis [4], differential sensitivity to chemotherapy [5], risk for recurrence [6], survival [7], malignant phenotype [8], and metastasis [9]. Although considerable variability in the composition of gene signatures was observed in these studies, they provided evidence for subsets within HNSCCs, which are possibly due to differences in molecular pathogenesis that affect malignant potential. However, the transcriptional regulatory mechanisms that control the heterogeneous and shared patterns of gene expression profiles observed, and their relationship to malignant phenotypes, are not well defined.

The transcriptional regulation of gene expression is mainly dependent on the composition of transcription factor binding site (TFBSs), and complex interactions among transcription factors and regulatory proteins that bind to gene promoters [10]. In murine and human squamous cell carcinoma (SCC), we and others have identified transcription factors that are inactivated or mutated (for instance, the tumor suppressor p53), or are constitutively activated (such as nuclear factor-κB [NF-κB], activator protein [AP]-1, signal transducer and activator of transcription [STAT]-3, and early growth response [EGR]1). These transcription factors have been independently implicated as tumor suppressor or oncogenic transcription factors that regulate the expression of individual genes related to phenotypic characteristics that are important in cancer development.

Among these transcription factors, p53 has been implicated as a master regulator of genomic stability, cell cycle, apoptosis, and DNA repair [11,12]. Mutation or silencing of the *p53 *gene is an important molecular event in tumorigenesis, which has been associated with nearly 50% incidence among all cancers [13-15], including HNSCC [16-20]. NF-κB is a nuclear transcription factor that is activated in HNSCCs and other cancers. We and others have shown that constitutive activation of NF-κB1/RelA is among the important factors that control expression of genes that regulate cellular proliferation, apoptosis, angiogenesis, immune and proinflammatory responses, and therapeutic resistance in HNSCCs [21-26] and other cancers [27-29]. AP-1, STAT3, and EGR1 are considered important transcription factors that are involved in regulating gene expression in human cancers, including HNSCCs. Constitutive activation of AP-1 and STAT3 appear to be important factors for tumor cell proliferation, survival, and angiogenesis *in vitro *or *in vivo *[21,24,29-34]. EGR1 is a zinc-finger transcription factor that is rapidly and transiently induced in response to a number of stimuli, including growth factors, cytokines, and mechanical stresses [35,36].

The study of regulatory controls involving multiple transcription factors for clustered gene expression obtained from microarray data meets with many experimental challenges. In a previous study of step-wise progression of murine SCCs, we combined gene expression profiling data with a bioinformatic analysis of promoter TFBSs and ontology limited to NF-κB regulated genes, and provided evidence that this transcription factor is one of the critical regulatory determinants of expression of multiple genes and malignant phenotype [37,38]. However, this approach involving analysis of a single pathway and this TFBS appears far from providing a complete explanation for the heterogeneity and multiplicity of genes expressed in clusters in HNSCCs and other cancers, or associated differences in phenotypic and biologic behavior observed. Identification of common TFBSs in gene clusters through *in silico *analysis can provide a framework for further elucidating the network and complex interactions of regulatory mechanisms that are involved in gene expression in cancer [39,40].

In the present study, microarray combined with computational prediction was utilized to define gene expression patterns and putative TFBSs for genes that are differentially expressed among ten HNSCC cell lines and nonmalignant keratinocytes. The differentially expressed microarray profiles classified subsets of HNSCC cells related to differences in p53 genotype, protein expression, and unique gene signatures. The potential relationship of novel gene expression signatures and prevalence of TFBSs for p53, NF-κB, AP-1, STAT3, and EGR1 were identified, and novel transcription regulatory modules for specific gene clusters were predicted. The predicted results were then validated by real-time reverse transcription (RT)-polymerase chain reaction (PCR) and chromatin immunoprecipitation (ChIP) assay. Our study suggests that integration of genome-wide microarray profiling and computational analyses is a powerful way to identify gene signatures as determinants for cancer heterogenicity and malignant phenotypes, and their underlying regulatory control mechanisms.

## Results

### Identification of novel gene clusters in University of Michigan SCC cells with different p53 status by cDNA microarray expression profiling

**Figure 1:**
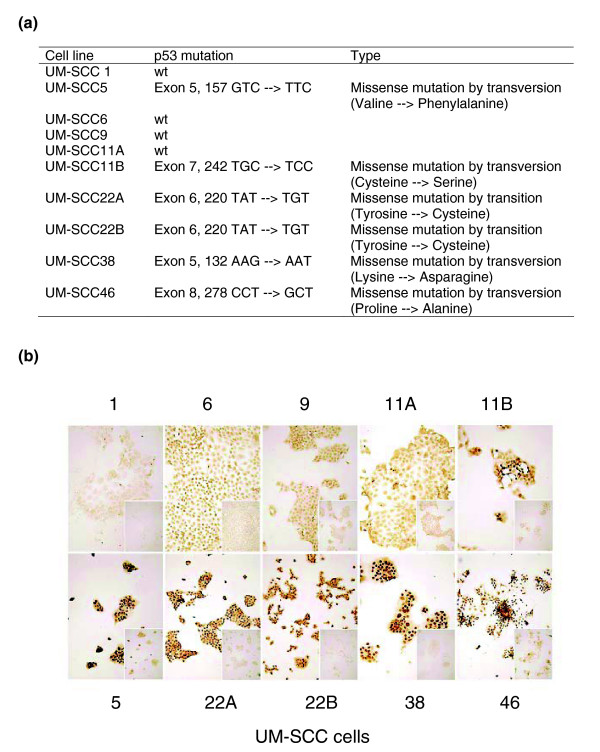
*p53 *genotype and protein expression in UM-SCC cell lines. **(a) **The *p53 *genotype of ten University of Michigan series head and neck squamous cell carcinoma (UM-SCC) cell lines was analyzed by two-directional sequencing of four to nine exons. **(b) **Immunohistochemistry for p53 was performed on the UM-SCC cell lines using anti-p53 monoclonal antibody (DO-1, clone), and the panels were segregated according to minimal or weaker staining pattern typical for wild-type p53 (upper panels, except UM-SCC 11B) and strong nuclear staining typical for mutant p53 status of cells (lower panels). The cells stained with the isotype control primary antibody as negative control are presented in the small pictures located at the lower right corner of each image. The pictures were taken at a magnification of 100×.

cDNA microarray analysis was performed using a panel of ten HNSCC cell line series from the University of Michigan (UM-SCC), derived from eight patients with aggressive HNSCC (survival <2 years), and representing a distribution of different anatomic sites (Table 1). Many of the molecular alterations and biologic characteristics of these UM-SCC cell lines have been confirmed to reflect those identified in HNSCC tumors from patients in laboratory and clinical studies. These include the roles of activation of epidermal growth factor receptor, IL-1, and IL-6 signal transduction pathways; altered activation of transcription factors p53, NF-κB, AP-1, and STAT3; expression of cytokines and other genes; and variation in radiation and chemosensitivity [5,21-26,30-33,41-43]. The *p53 *mutation and expression status of UM-SCC cells lines were evaluated using bidirectional genomic sequencing of exons 4 to 9 (Figure 1a), and confirmed with immunocytochemistry using monoclonal antibody to p53 (DO-1 clone) (Figure 1b). No mutation was detected in those exons in four cell lines, namely UM-SCC 1, 6, 9, and 11A. Mutation of p53 was detected in five cell lines, namely UM-SCC 5, 22A, 22B, 38, and 46 (Figure 1a). A mutation was also detected in UM-SCC 11B cells, but immunocytochemistry of p53 protein suggested there might be a mixed population of UM-SCC 11B cells with a heterogeneous expression pattern for nuclear p53 protein (Figure 1b). The findings regarding *p53 *mutation in UM-SCC 1, 5, 6, 11B, and 46 cells are consistent with a previous report by Bradford and coworkers [41].

**Table 1:** Tumor, treatment, and outcome characteristics of patients providing human SCC cell lines

Cell line	Age (years at diagnosis)	Sex	Stage	TNM	Primary site	Specimen site	Prior therapy	Status	Survival (months)
UM-SCC 1	72	M	I	T1N0M0	FOM	Local recur	R	DWOD	15
UM-SCC 5	59	M	III	T2N1M0	Supraglottic larynx	Pri bx	S	DOD	8
UM-SCC 6	37	M	II	T2N0M0	Tongue	Pri bx	N	LTF	
UM-SCC 9	72	F	II	T2N0M0	Tonsil/BOT	Local recur	R	DOD	15
UM-SCC 11A	65	M	V	T2N2aM0	Hypopharynx	Pri bx	N	DOD	14
UM-SCC 11B						Pri resect	C		
UM-SCC 22A	59	F	III	T2N1M0	Hypopharynx	Pri bx	N	DOD	10
UM-SCC 22B						LN met	N		
UM-SCC 38	60	M	IV	T2N2aM0	Tonsil/BOT	Pri	N	DOD	11
UM-SCC 46	57	F	III	No TMN Given	Suprglottic larynx	Local recur	R, S	DOD	6

The clinical information was kindly provided by Drs Thomas E Carey and Carol R Bradford, and some information was previously presented in the literature. 'Primary sites' refers to the origin of the primary tumor. 'Specimen site' refers to origin of tissue used to establish cultures. 'Prior therapy' refers to therapy given before the specimen used for culture was obtained. 'Survival' represents time in months from diagnosis to last follow up. BOT, base of tongue; bx, biopsy; C, chemotherapy; DOD, died with disease; DWOD, died without disease; F, female; FOM, floor of mouth; LN, lymph nodes; LTF, lost to follow-up; M, male; met, metastasis; N, none; NED, no evidence of disease; Pri, primary tumor site; R, radiation; recur, recurrence; resect, surgical resection specimen; SCC, squamous cell carcinoma; S, surgery; TNM, tumor-node-metastasis (staging system); UM-SCC, University of Michigan series head and neck squamous cell carcinoma.

Gene expression profiles were determined using a 24,000 element cDNA microarray by comparing 10 UM-SCC cell lines with four cultured primary human keratinocyte (HKC) lines as normal controls. The expression of 9,273 of 12,270 evaluable known genes was submitted for principal components analysis and hierarchical clustering [44]. Both methods grouped UM-SCC 11B together with its parental cell line UM-SCC 11A, as well as the other UM-SCC cells with wild-type p53, and these findings were statistically significant (*P *< 0.001, class prediction analysis; BRB-Array Tools [45]). Based on mixed p53 protein staining and wild-type p53 associated gene expression pattern, we classified UM-SCC 11B cells with the wild-type p53 group as having a 'wild-type p53-like' expression pattern.

**Figure 2:**
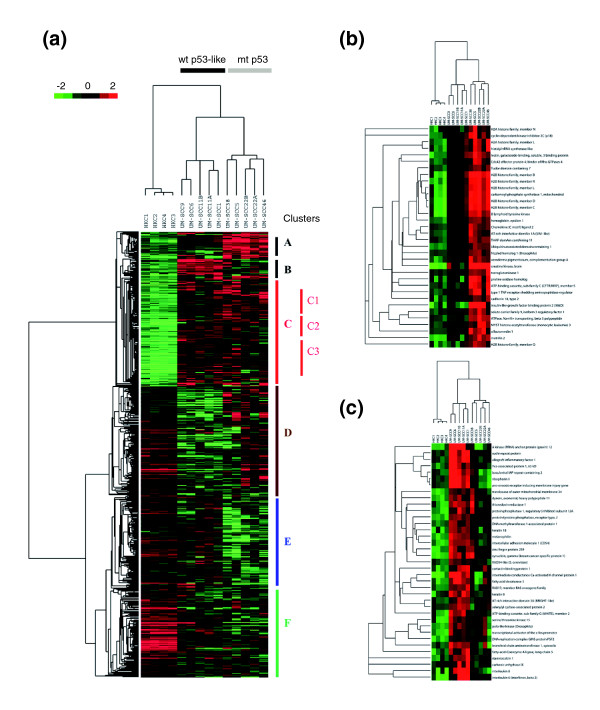
Hierarchical clustering analysis of differentially expressed genes in UM-SCC cells. A total of 1,011 differentially expressed genes was extracted from 24,000 cDNA microarray database, based on twofold and greater difference among human normal kerintinocytes (HKCs), UM-SCC cells with wild-type p53-like expression pattern, mutant p53 or wild-type + mutant p53 status (*t*-test score at *P *< 0.05, two-tailed). The hierarchical clustering tree was generated using Java Treeview [107]. Four HKCs were grouped on the left, and five UM-SCC cell lines with wild-type p53-like expression pattern were grouped together in the middle, and five UM-SCC cell lines with mutant p53 were grouped to the right, respectively. Over-expressed genes are indicated by red and under-expressed genes by green; and the expression level is proportional to the brightness of the color (see color bar). **(a) **Entire hierarchical clustering tree included three upregulated clusters (A, B and C [including subclusters C1 to C3]) and three downregulated clusters (D, E and F). **(b) **Cluster A consisted of 34 genes. **(c) **Cluster B consisted of 37 genes. mt, mutant; wt, wild-type.

Next, we studied a total of 1,011 genes that exhibited twofold or greater differences in gene expression when comparing HKCs with all UM-SCC, or comparing UM-SCC cells with either wild-type p53-like (UM-SCC cell lines 1, 6, 9, 11A, and 11B) or mutant p53 expression patterns (UM-SCC cell lines 5, 22A, 22B, 38, and 46; Figure 2a). The 1,011 genes, including 371 over-expressed and 640 under-expressed genes, were subjected to hierarchical clustering, as shown in Figure 2a. The expression profile of 1,011 genes clustered all samples into three groups, namely HKCs, UM-SCC wild-type p53-like, and UM-SCC with mutant p53 (Figure 2a). Six major clusters (A to F) of differentially expressed genes were identified, with most over-expressed genes included in two distinct clusters, A and B, and three subclusters within cluster C (subclusters C1, C2, and C3) on the top portion of the expression tree (Figure 2a).

The unique gene signatures of clusters A and B consisted of 34 and 37 genes (Figure 2b,c and Table 2), respectively. We used the mixed model based F-test to examine the statistical difference of gene expression within clusters among HKCs and UM-SCC cell lines with different expression patterns. Within both cluster A and cluster B genes, a significant difference in gene expression (probability, *Pr [F] *< 0.001) was observed when comparing the two groups of UM-SCC cells. A significant difference was also observed when comparing HKCs with UM-SCC cells with mutant p53 in cluster A, and comparing UM-SCC with the wild-type p53-like expression pattern in cluster B. Thus, cluster A genes were over-expressed in UM-SCC cells with mutant p53 (Figure 2b), whereas cluster B genes were over-expressed in UM-SCC cells with wild-type p53-like expression pattern (Figure 2c).

**Table 2:** Putative transcription factor binding sites of clusters A and B over-expressed in HNSCC

Gene name	Gene description	RefSeq	Orthlog^a^	Number of TFBSs predicted^b^					Functional annotation^c^
					
				p53	NF-κB	AP-1	STAT3	EGR1	
Cluster A									
*ABCC5*	ATP-binding cassette, subfamily C (CFTR/MRP), member 5	NM_005688	hmr^a^				1 (hmr)		Transport
*ARID1A*	AT rich interactive domain 1a (SWI-like)	NM_006015	hmr	1 (hmr)				5 (hmr)	Regulation of metabolism
*ARTS-1*	Type 1 TNF receptor shedding aminopeptidase regulator	NM_016442	hmr	1	1		1	1	Catabolism
*ATP1B3*	ATPase, Na^+^/K^+ ^transporting, beta 3 polypeptide	NM_001679	hmr		3			7	Transport
*BLK*	B lymphoid tyrosine kinase	NM_001715	hmr			1			Signal transduction
*CDC42EP4*	cdc42 effector protein 4; binder of Rho GTPases 4	NM_012121	h	1	2		2		Regulation of cell shape
*CDH18*	Cadherin 18, type 2	NM_004934	h						Cell adhesion
*CDKN2C*	Cyclin-dependent kinase inhibitor 2C (p18)	NM_078626	hmr	3	1				Cell proliferation; cell cycle
*CKB*	Creatine kinase, brain	NM_001823	hmr		3			8	Creatine kinase activity
*CPS1*	Carbamoyl-phosphate synthetase 1, mitochondrial	NM_001875	hmr	2 (hmr)	1	1 (hmr)			Amino acid metabolism
*FZD1*	Frizzled homolog 1 (*Drosophila*)	NM_003505	h		1			4	Signal transduction
*HARSL*	Histidyl-tRNA synthetase-like	NM_012208	hm		2		1	1	Amino acid metabolism
*HBE1*	Hemoglobin, epsilon 1	NM_005330	hr		1		1		Transport
*HIST1H2AC*	H2A histone family, member L	NM_003512	h		1				Chromosome organization and biogenesis
*HIST1H2AM*	H2A histone family, member N	NM_003514	h	3					Chromosome organization and biogenesis
*HIST1H2BC*	H2B histone family, member L	NM_003526	h		1				Chromosome organization and biogenesis
*HIST1H2BD*	H2B histone family, member B	NM_138720	hr	1	1				Chromosome organization and biogenesis
*HIST1H2BJ*	H2B histone family, member R	NM_021058	h				1	1	Chromosome organization and biogenesis
*HIST1H2BL*	H2B histone family, member C	NM_003519	h	1					Chromosome organization and biogenesis
*HIST1H2BN*	H2B histone family, member D	NM_003520	h	2			2		Chromosome organization and biogenesis
*HIST2H2BE*	H2B histone family, member Q	NM_003528	h	3	1		1	1	Chromosome organization and biogenesis
*IGFBP2*	Insulin-like growth factor binding protein 2 (36 kDa)	NM_000597	hmr		1 (hr)			4 (hmr)	Regulation of cell growth
*LGALS3BP*	Lectin, galactoside-binding, soluble, 3 binding protein	NM_005567	hmr		1		2 (hmr)		Cell adhesion
*MATN2*	Matrilin 2	NM_002380	h	1					Extracellular matrix assembly
*MYST3*	MYST histone acetyltransferase (monocytic leukemia) 3	NM_006766	hmr		5 (hmr)			6 (hmr)	DNA packaging
*OLFM1*	Olfactomedin 1	NM_014279	hmr	1	1		2	4 (hmr)	Morphogenesis
*PRODH*	Proline oxidase homolog	NM_016335	h						Amino acid metabolism
*SLC9A3R1*	Solute carrier family 9, isoform 3 regulatory factor 1	NM_004252	hmr		1			3 (hr)	Signal transduction
*TDRD7*	Tudor domain containing 7	NM_014290	hmr	1	1 (hmr)			5 (hmr)	Protein amino-terminus binding
*TGM1*	Transglutaminase 1	NM_000359	hmr	2		1		1	Morphogenesis; cell proliferation
*THAP11*	THAP domain containing 11	NM_020457	h					3	DNA binding, ion binding
*UBADC1*	Ubiquitin associated domain containing 1	NM_016172	hmr	2 (hmr)				7 (hmr)	Protein ubiquitination
*XCL1*	Chemokine (C motif) ligand 2	NM_002995	h	2					Signal transduction
*XPA*	Xeroderma pigmentosum, complementation group A	NM_000380	hmr	2		1		2 (hmr)	DNA repair
									
Cluster B									
*ABCG2*	ATP-binding cassette, subfamily G (WHITE), member 2	NM_004827	h		3	1		2	Transport
*ACSL5*	Fatty-acid-coenzyme a ligase, long-chain 5	NM_016234	hmr		1 (hmr)			1	Fatty acid metabolism
*AIF1*	Allograft inflammatory factor 1	NM_001623	hmr						Inflammatory response; cell cycle
*AKAP12*	A kinase (PRKA) anchor protein (gravin) 12	NM_005100	hmr	1	2			2	Signal transduction
*ARID3A*	AT rich interactive domain 3A (BRIGHT-like)	NM_005224	hmr	2 (hmr)	1			3	Regulation of transcription
*BCAT1*	Branched chain aminotransferase 1, cytosolic	NM_005504	h		1	1		2	Cell cycle; amino acid metabolism
*BIRC2*	Baculoviral IAP repeat-containing 2	NM_001166	h		2	2	1	2	Antiapoptosis; signal transduction
*CA9*	Carbonic anhydrase IX	NM_001216	hmr		1 (hmr)	1 (hmr)			One-carbon compound metabolism
*CAP2*	Adenylyl cyclase-associated protein 2	NM_006366	hm			1			Signal transduction
*CROC4*	Transcriptional activator of the c-fos promoter	NM_006365	h	1					Cell proliferation
*DMAP1*	DNA methyltransferase 1-associated protein 1	NM_019100	hmr		3 (hr)	1			Regulation of transcription
*DNAH11*	Dynein, axonemal, heavy polypeptide 11	NM_003777	h					4	Transport
*FADS3*	Fatty acid desaturase 3	NM_021727	hmr		3			5 (hr)	Fatty acid metabolism
*ICAM1*	Intercellular adhesion molecule 1 (CD54)	NM_000201	hmr		2 (hmr)	1	1 (hmr)	3	Cell adhesion
*IL6*	Interleukin 6 (interferon, beta 2)	NM_000600	hmr	1 (hm)	1 (hmr)	1 (hmr)		1	Signal transduction; inflammatory response
*IL8*	Interleukin 8	NM_000584	h		1	1	1		Signal transduction; inflammatory response
*KCNN4*	Intermediate conductance Ca-activated K channel protein 1	NM_002250	hmr	1	3 (hmr)	1 (hmr)			Transport
*KRT18*	Keratin 18	NM_199187	h	1	1				Structural molecule activity
*KRT8*	Keratin 8	NM_002273	hr	2	2	1 (hr)		2	Structural molecule activity
*MLPH*	Melanophilin	NM_024101	h		1			2	Transport
*Pfs2*	DNA replication complex GINS protein PSF2	NM_016095	h	1				5	DNA metabolism
*PLK1*	Polo-like kinase (*Drosophila*)	NM_005030	hmr		1		1	1 (hm)	Metabolism; cell proliferation
*PORIMIN*	Pro-oncosis receptor inducing membrane injury gene	NM_052932	h		1			5	Oncosis-like cell death
*PPP1R12A*	Protein phosphatase 1, regulatory (inhibitor) subunit 12A	NM_002480	hmr		1			5 (hmr)	Regulation of organismal physiological process
*PTPRJ*	Protein tyrosine phosphatase, receptor type, J	NM_002843	h		1			8	Signal transduction
*RAB17*	RAB17, member RaS oncogene family	NM_022449	h		1			2	Signal transduction
*RAD54L*	RAD54-like (*S. cerevisiae*)	NM_003579	hmr			1		7	DNA repair; cell cycle
*RPN2*	Ribophorin II	NM_002951	hmr		1	1	1	1	Protein metabolism
*SHANK2*	Cortactin binding protein 1	NM_012309	hmr		2		2	4	Signal transduction
*SNCG*	Synuclein, gamma (breast cancer-specific protein 1)	NM_003087	hmr					1	Pathogenesis
*SRPX2*	Sushi-repeat protein	NM_014467	hmr			2 (hmr)			Electron transport
*STC1*	Stanniocalcin 1	NM_003155	hmr	1			3 (hmr)		Signal transduction
*STK6*	Serine/threonine kinase 15	NM_198433	hmr				1		Cell cycle
*TOMM34*	Translocase of outer mitochondrial membrane 34	NM_006809	hmr		3 (hmr)			2 (hmr)	Protein metabolism
*TXNRD1*	Thioredoxin reductase 1	NM_003330	hmr		1	2		1 (hmr)	Signal transduction
*YAP1*	Yes-associated protein 1, 65 kD	NM_006106	hmr		1			11(hmr)	Signal transduction
*ZNF239*	Zinc finger protein 239	NM_005674	h				1		Regulation of transcription

Shown are numbers of transcription factor binding sites (TFBSs) from p53, nuclear factor-κB (NF-κB), activator protein (AP)-1, signal transducer and activator protein (STAT)3, and early growth response (EGR)1 in clusters A and B over-expressed in head and neck squamous cell carcinoma (HNSCC). TFBSs were predicted using Genomatix Suite 3.4.1 [108]. ^a^Orthologous promoter sets are indicated by single-letter abbreviations (h, human; m, mouse; r, rat). ^b^Values are presented as number of TFBSs in proximal region of promoters. The average length of these promoters was adjusted to approximately 600 base pairs (bp): about 500 bp upstream and about 100 bp downstream. Letters in the parentheses refer to conserved TFBSs identified among human, mouse, or rat using multiple sequence alignment of DiAlign TF of Genomatix Suite 3.4.1. ^c^From Gene Ontology Annotation using Onto-Express [46], AmiGo [106], and National Center for Biotechnology Information [107]. TNF, tumor necrosis factor.

In addition to genes of clusters A and B, we defined another group of over-expressed genes, namely cluster C, including three subclusters C1, C2, and C3 (Figure 2a and Additional data file 1). Overall, cluster C contained 240 genes that were over-expressed by 10 cancer cell lines when compared with HKCs (*Pr [F] *< 0.001). However, two of the subclusters (C1 and C2) identified exhibited a degree of differential expression in UM-SCC cells similar to the various p53-associated expression patterns (Additional data file 1).

### Gene Ontology annotation revealed the unique nature of clustered genes

To determine the functional classification of the various gene clusters, we conducted Gene Ontology (GO) annotation using Onto-Express, which constructs statistically significant functional profiles from a set of genes [46]. Additional data file 2 shows functional categories that are significantly enriched in the six clusters. The top categories of GO biologic processes in cluster A were nucleosome assembly, chromosome organization, and biogenesis. These included genes involved in regulation of chromosome structure or function (such as H2B histone family B, C, D, R, L and Q, and H2A histone family L and N), transport (such as *MYST3*, *ABCC5*, *ATP1B3*, and *HBE1*), and DNA repair (such as *XPA*). The main GO molecular function was DNA binding, including eight genes in histones H2A and H2B, and *MYST3*, *THAP11 *and *ARID1A *(Table 2 and Additional data file 2).

In contrast to cluster A, the top ranked GO biologic processes in cluster B belonged to signal transduction (such as cell-cell signaling, cell surface receptor linked signal transduction), including *AKAP12*, *CAP2*, *IL6*, *IL8*, *RAB17*, *SHANK2*, *STC1*, *PTPRJ*, *TXNRD1*, and *YAP1 *(Table 2 and Additional data file 2). Other enriched functional categories included cell cycle (*AIF1*, *BCAT1*, *RAD54L*, and *STK6*), regulation of transcription (*ARID3A*, *DMAP1*, and *ZNF239*), cell proliferation and apoptosis (*CROC4*, *BIRC2*, *PLK1*, and *PORMIN*), adhesion (*ICAM1*), and structural proteins related to tumor progression (*KRT8 *and *KRT18*; Table 2 and Additional data file 2). Interestingly, several genes in this cluster or their homologs involved in angiogenesis and inhibition of apoptosis have previously been associated with metastatic tumor progression in murine SCC or human HNSCC (*IL6*, *IL8*, *YAP1*, and *BIRC2*) [9,22-24,30,33,37,38,47-49], and shown to be regulated by NF-κB [22-25,37,49].

Genes in cluster C exhibited annotations for DNA replication, ubiquitin cycle, cell division, and oxidoreductase and catalytic activities. The gene list and ontology of the subclusters in C are presented in the Additional data files 1 and 2, respectively. Several genes in subclusters C1 and C2 exhibited weaker clustering but similar functions as those in cluster A or B. In subcluster C1, in which over-expressed genes were mainly found in UM-SCC cells with mutant p53 as in cluster A, there are two additional genes identified that encode proteins involved in chromosome structure and functions (*HIST1H2AL *and *HDAC5*). Other genes previously associated with cancer included a member of epidermal growth factor receptor family (*ERBB3*); a target gene of p53/p63 (*IGFBP3*); and a gene whose product is involved in calcium storage and signaling (*CALR*). In subcluster C2, in which over-expressed genes were found in UM-SCC cells with wild-type p53-like expression pattern as in cluster B, another apoptosis related gene (*BAG2*), genes encoding signal-related molecules (*MYBL2 *and *UBE2C*) and a cell cycle related molecule (*CCNB2*) were identified (Additional data file 1). The rest of the cluster C genes were over-expressed by more than half of ten UM-SCC cells when compared with HKCs. Several genes encoding protein products that are important in cancer and have functions related to cell cycle, growth, DNA replication and protein translation (such as *CCND1*, *TOP2A*, *TOPBP1*, *TFRC*; three members of H4 histone family [*HIST1H4C*, *HIST1H4B*, and *HIST1H4E*]; and *EIF4G1*). Some genes encode proteins with functions related to signal transduction (*PIK3R3*, *MAPK8IP1*, and *GATA2*), and one gene encodes a protein that regulates tumor invasion and metastasis (*TIMP2*).

Genes downregulated in UM-SCC cells were included in cluster D, which represents functional categories that are involved in epidermis development, cell adhesion, and cell-cell signaling (Additional data file 2). Downregulated cluster E genes included those encoding molecules with functions in other signal pathways, cell cycle, calcium ion regulation, and actin binding activities (Additional data file 2). The categories over-represented among the downregulated genes in cluster F included cell adhesion, differentiation, and morphogenesis (Additional data file 2). A more detailed analysis of the downregulated genes will be presented elsewhere (Yan, unpublished data).

### Over-representation of binding sites of five transcription factors associated with the unique gene clusters in UM-SCC cells

Based on the gene expression profiling data, we hypothesized that transcriptional regulation by multiple transcription factors may be key elements that contribute to the expression of unique gene clusters. To test this hypothesis, *in silico *computational analyses were performed to determine whether dominant *cis-*regulatory elements are present in the proximal promoter region of over-expressed genes. We evaluated five transcription factors that were previously found to be altered and functionally important in HNSCCs and other cancers, including p53, NF-κB, AP-1, STAT3, and EGR1. We compared the frequencies of their binding sites with those from vertebrate promoters from the Genomatix promoter database (GPD), which consists of information from human, mouse, and rat. The five transcription factors examined have been shown by our laboratory and others to contribute to regulation of individual gene expression with functional importance in cancer, such as cell proliferation, cell cycle, apoptosis, DNA repair, and angiogenesis [21,22,24,36-38,50-53].

**Figure 3:**
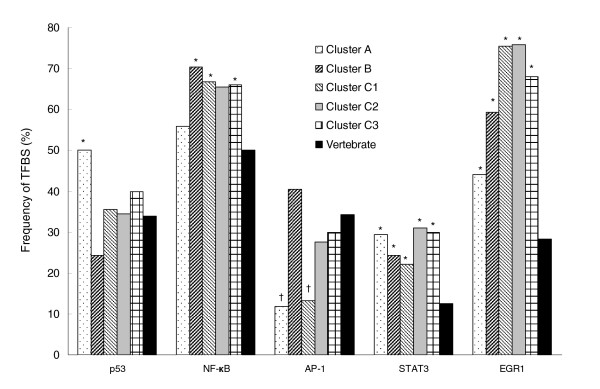
Frequency of putative TFBSs in proximal regions of promoters. The promoter sequences were extracted from the over-expressed genes in clusters A and B, and subclusters C1 to C3 in UM-SCC cells using Genomatix Suite 3.4.1. The average length of these promoters was adjusted to approximately 600, including about 500 base pairs upstream and about 100 base pairs downstream from the transcription start site. The promoter sequences from vertebrates represented 159,505 promoters, including 55,207 from human, 69,108 from mouse, and 35,190 from rat in Genomatix promoter database. The *P *value of transcription factor binding site (TFBS) frequency in a given cluster was calculated by MatInspector of Genomatix Suite 3.4.1. *Significantly increased frequencies of putative binding motifs on promoter regions of clustered genes when compared with the vertebrate promoters with a randomly drawn sample of the same size (*P *< 0.05). ^†^Significantly lower frequency of the activator protein (AP)-1 binding motif when compared with the vertebrate promoters. EGR, early growth response; NF-κB, nuclear factor-κB; STAT, signal transducer and activator of transcription.

Table 2 shows a list of genes included in clusters A and B and corresponding binding sites for the five transcription factors in proximal promoter regions that are predicted with high probability. The detailed location and sequences of the putative TFBSs are shown in Additional data file 3. Significant differences in the prevalence of predicted TFBSs were observed for genes from different clusters when compared with vertebrate promoters (Figure 3). In cluster A, putative p53 binding sites were detected in 50% of the 34 gene promoters, which is significantly higher than observed in vertebrate promoters (*P *< 0.05; Figure 3). Conversely, predicted NF-κB binding sites were observed in about 66% to 70% of the promoters in clusters B and C, which was significantly more than in vertebrate promoters. There was no significant difference in the prevalence of NF-κB binding sites between the promoters of cluster A and vertebrates. There were also differences in the prevalence of TFBSs predicted between different clusters. For example, the p53 binding motif was significantly greater in cluster A than cluster B (χ^2 ^analysis; *P *< 0.05), and the greatest frequency of NF-κB binding sites was observed in cluster B (26/37 [70%]; Figure 3). There were significantly fewer genes with AP-1 binding sites in cluster A and subcluster C1 (12% and 13%, respectively) compared with vertebrate promoters (Figure 3). A relatively higher frequency of AP-1 binding sites was observed in cluster B genes when compared with frequencies in cluster A and subcluster C1 genes (χ^2 ^analysis; *P *< 0.01). In contrast, a relative increase in prevalence of STAT3 and EGR1 binding sites was observed and distributed among all of the upregulated clusters relative to vertebrate promoters (Figure 3), with increasingly higher frequencies of EGR1 motifs detected in clusters B and C (60% to 76%; using Genomatix matrix EGR1.02).

### The orthologous promoters and conserved transcription factor binding sites predict increased likelihood of functional co-regulation of clustered genes

The likelihood of functionality of a predicted TFBS can be examined by determining its conservation at the sequence level. To determine the potential conservation of the predicted TFBSs, the orthologous promoter regions of genes in clusters A and B were examined by searching their conservation at the sequence level among vertebrates (human, mouse, and rat) using the comparative genomics analysis feature of Genomatix Suite 3.4.1. Orthologous promoter sets were found in 19 and 24 genes of clusters A and B, respectively (Table 2; Ortholog). Among 17 genes containing predicted p53 binding sites in cluster A, 10 out of 17 (59%) were identified in the orthologous promoter regions. In this cluster, the predicted prevalence of binding sites falling in the orthologous promoter regions were 63% for NF-κB, 60% for AP-1, 100% for STAT3, and 76% for EGR1. Similarly, in cluster B, the prevalence of binding sites falling in orthologous promoters were 65% for NF-κB, 67% for p53 and AP-1, 73% for STAT3, and 64% for EGR1. These levels of conservation indicated that the majority of predicted TFBSs falling in the orthologous promoter regions were likely selected favorable for growth or survival during evolution. Interestingly, although expression of histone H2A and H2B gene members were predominant in cluster A, only a rat orthologous promoter was found in *HIST1H2BD *among the eight histone genes (Table 2).

**Figure 4:**
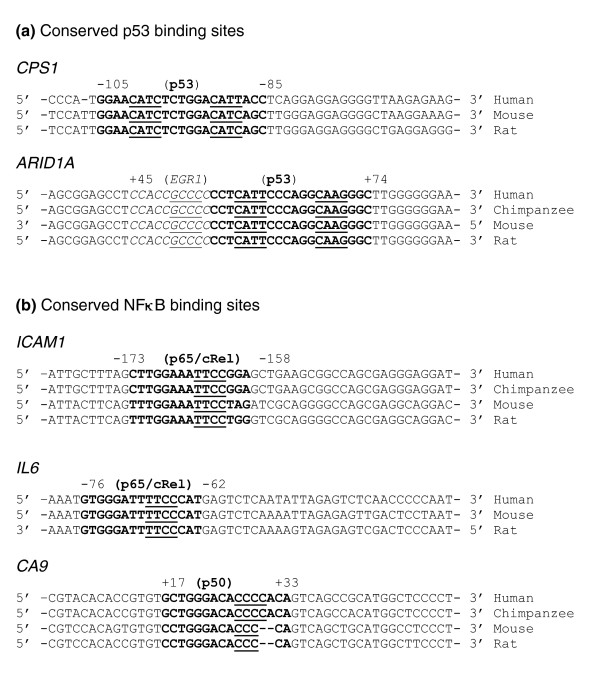
Predicted conserved p53 and NF-κB binding sites in proximal promoter regions of five representative genes from clusters A and B. The search for conserved TFBS was carried out by multiple sequence alignment of each promoter set using DiAlignTF of Genomatix Suite 3.4.1. The promoter region included about 500 base pairs upstream and about 100 base pairs downstream from the transcription start site (TSS) among human, chimpanzee, mouse, and rat. **(a) **The conserved p53 binding motifs were present in two gene promoters from cluster A (*CPS1 *and *ARID1A*), and **(b) **conserved nuclear factor-κB (NF-κB) binding motifs were present in three gene promoters from cluster B (*ICAM1*, *IL6*, and *CA9*). Letters in bold are the predicted binding sites of p53 or NF-κB, letters in italic are early growth response (EGR)1 binding sites, and letters underlined denote the core conserved sequence. The numbers showed predicted transcription factor binding site (TFBS) position from the TSS of human sequences, where negative positions were upstream of the TSS and positive ones were downstream from the TSS.

The conserved TFBSs among the orthologous promoter sets were further investigated by multiple sequence alignment using DiAlignTF [54]. Conserved p53 binding sites were found in three genes of cluster A (*ARID1A*, *CPS1*, and *UBADC1*) and two genes of cluster B (*IL6 *and *ARID3A*; Table 2). The conservation of NF-κB binding sites was observed in more genes, including *LGALS3BP*, *MYST3 *and *TDRD7 *in cluster A, and *ACSL5*, *CA9*, *DMAP1*, *ICAM1*, *IL6*, *KCNN4*, and *TOMM34 *in cluster B. Additionally, the binding sites of AP-1, STAT3, and EGR1 were conserved in 6, 4, and 14 gene promoters, respectively (Table 2). Next, we identified five representative gene promoters from either cluster A or B genes, which contained conserved p53 or NF-κB binding motifs among human, chimpanzee, mouse, and rat (Figure 4). The core sequence (underlined) of a transcription factor matrix represents the most highly conserved and consecutive positions of this matrix. In promoters of both *CPS1 *and *ARID1A *from cluster A genes, the predicted p53 binding sites were similar to Genomatix and TRANSFAC p53 matrix consensus sequence GGACATGCCGGGCATGTCY (Figure 4a). The p53 binding site of *ARID1A *promoter was located 55 to 74 base pairs (bp) downstream from the transcriptional start site and overlapped with a EGR1 binding site. Known NF-κB sites in *IL6 *and *ICAM1 *promoters are conserved with about 90% matrix similarity to the five matrices for the NF-κB family, including p65 and cRel (Figure 4b and Additional data file 3). Another conserved NF-κB site in the promoter of gene *CA9 *exhibited 85% to about 90% similarity to two NF-κB matrices of the family including p50, indicating that these sites are more likely to be functional in a biologic context.

### Novel transcription factor regulatory modules associated with p53 or nuclear factor-κB in promoters of clustered genes

**Figure 5:**
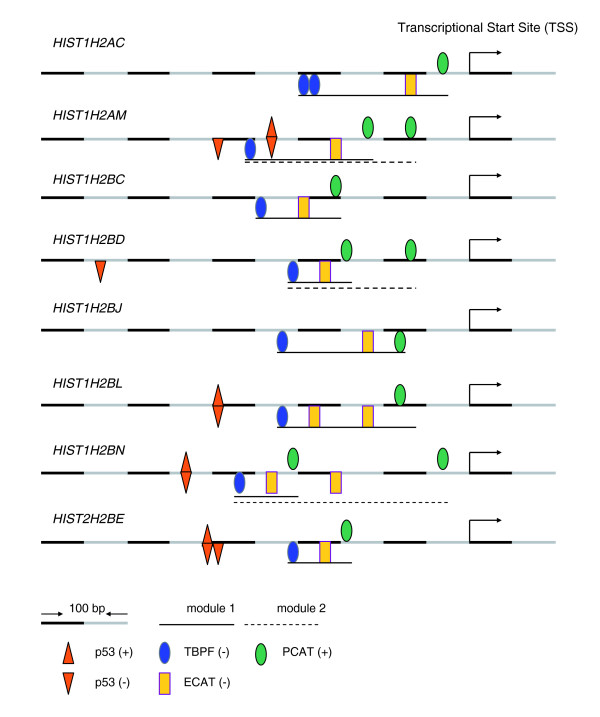
Transcription regulatory module containing multiple transcription factors in eight histone gene promoters from cluster A. Using FrameWorker of Genomatix Suite 3.4.1, eight promoter regions of histone genes (two H2A and six H2B) from cluster A were used to predict regulatory modules including TBPF (TATA-binding protein factors), ECAT (enhancer of CCAAT binding factors), or PCAT (promoter of CCAAT binding factors). p53 binding motifs were also displayed. '(+)' and '(-)' refer to strand direction of transcription factor binding motifs.

Because we observed that several transcription factors are often co-activated in HNSCCs, we hypothesized that the clustered gene expression could be co-regulated by multiple transcription factors [55]. These transcription factors are expected to be structured and coordinated tightly together, form a functional unit or so-called transcription factors module, and play roles in regulating gene expression. To obtain evidence for this hypothesis, we used FrameWorker of Genomatix Suite 3.4.1 to define promoter models. Based on the promoter modeling, we identified the putative regulatory modules of TFBSs in the clustered genes that were over-expressed by UM-SCC cells. Two co-regulated gene groups were selected for this analysis, which included 17 genes with p53 binding sites in cluster A and 26 genes with NF-κB binding sites in cluster B. In cluster A genes with p53 binding sites, putative models containing three and four transcription factors were present with scores of high selectivity (Table 3), indicating that such models are enriched to a greater extent in cluster A genes than in genes randomly selected from the whole human genome. All eight transcription factor models contained p53-TBPF (TATA-binding protein factors) associated with either CREB (cAMP-responsive element binding proteins; 4/8) or PCAT (promoter of CCAAT-binding factors; 2/8), suggesting the possible functional relationships or co-regulatory mechanisms mediated by these transcription factors. These transcription factor modules were over-represented in the proximal promoter regions of several genes, including *CPS1 *in all eight models, and *HIST1H2AM*, *HIST1H2BE*, and *HIST1H2BL *in six to seven models. In addition to genes with p53 binding motifs, we also identified a putative module of TBPF-ECAT (enhancer of CCAAT binding factors)-PCAT that was present on 100% promoter regions within eight histone H2A or H2B genes (Figure 5), which is in contrast to the low frequency (0.47%) observed in the entire human promoter database. The putative p53 binding sites found in the promoters of four histone genes, namely *HIST1H2AM*, *HIST1H2BE*, *HIST1H2BL*, and *HIST1H2BN*, were located within 100 bp of the TBPF-ECAT-PCAT module (Figure 5), which is consistent with a greater likelihood of regulatory interactions.

**Table 3:** Putative transcription factor models in clusters A and B over-expressed in HNSCC

Model	Model matches in the cluster	% of matches in the cluster^a^	% of hits in GPD^b^	Selectivity^c^
Selected genes from cluster A^d^				
**p53**-TBPF	HIST1H2AM, HIST2H2BE, HIST1H2BL, HIST1H2BN, ***CPS1*, XPA**, XCL1	41	4.39	9.4
**p53**-TBPF-CREB	HIST1H2AM, HIST2H2BE, HIST1H2BL, ***CPS1***, XCL1	29	0.68	43.3
**p53**-TBPF-PCAT	HIST1H2AM, HIST2H2BE, HIST1H2BL, HIST1H2BN, ***CPS1***	29	0.25	119.4
**p53**-TBPF-CREB-TBPF	HIST1H2AM, HIST2H2BE, HIST1H2BL, ***CPS1***, XCL1	29	0.19	151.8
**p53**-TBPF-PCAT-SORY	HIST2H2BE, HIST1H2BL, HIST1H2BN, ***CPS1***	24	0.02	1180.9
**p53**-TBPF-CREB-ECAT	HIST1H2AM, HIST2H2BE, HIST1H2BL, ***CPS1***	24	0.05	433.0
**p53**-TBPF-VBPF-TBPF	HIST2H2BE, HIST1H2BL, ***CPS1***, XCL1	24	0.06	371.1
**p53**-TBPF-CREB-CDXF	HIST1H2AM, HIST2H2BE, HIST1H2BL, ***CPS1***	24	0.04	541.2
				
Selected genes from cluster B^e^				
ETSF-**NFκB**	***TOMM34*, *DMAP1*, *ACSL5*, YAP1, AKAP12, PLK1, TXNRD1**, IL8, ABCG2, BIRC2, RAB17	42	17.21	2.5
**NFκB**-ETSF	***ICAM1*, *DMAP1*, YAP1, RPN2, AKAP12, SHANK2**, IL8, ABCG2, BIRC2, RAB17,	38	13.45	3.1
ZBPF-**NFκB**	***IL6*, *ICAM1*, *TOMM34*, *KCNN4*, *CA9*, FADS3, RPN2, SHANK2**, ABCG2, RAB17	38	12.76	3.0
EGRF-**NFκB**^f^	***ICAM1*, TOMM34**, *CA9*, *DMAP1*, *FADS3*, *KRT8*, ABCG2, MLPH,	31	15.71	2.0
**NFκB**-EGRF(+)^f^	***ICAM1*, *KCNN4*, YAP1, FADS3, RPN2, AKAP12, TXNRD1**, ABCG2	31	16.64	1.8
**NFκB**-EGRF(-)^f^	***ICAM1*, *TOMM34*, FADS3, TXNRD1**, ABCG2, MLPH, PORIMIN, PTPRJ	31	16.46	1.9
**NFκB**-STAT^f^	***ICAM1*, *TOMM34*, *DMAP1*, FADS3, KRT*8***, ABCG2, MLPH	27	9.57	2.8
SP1-ZBPF-**NFκB**	***IL6*, *ICAM1*, *KCNN4*, *CA9*, FADS3, KRT8**	23	4.02	5.7
**NFκB**-ZBPF-EGRF	**YAP1, TXND1, AKAP12, FADS3, PPP1R12A, RPN2**	23	6.75	3.4
ZBPF-**NFκB**-MAZF	***KCNN4*, *DMAP1*, YAP1, FADS3, TXNRD1**, ABCG2	23	2.45	9.4
**NFκB**-PAX5-ZBPF-ZBPF^g^	***DMAP1*, TXNRD1, FADS3**, ABCG2, BCAT1	19	1.46	13.2
CREB-ZBPF-**NFκB**-ETSF^g^	***IL6*, *TOMM34*, *KCNN4***, ABCG2, PTPRJ	19	0.35	54.7
NKXH-HOXF-CREB-**NFκB**-ETSF^g^	***IL6*, *TOMM34*, *DMAP1***, IL8	15	0.03	530.8
HNF1-HOXF-CREB-**NFκB**-ETSF^g^	***IL6*, *TOMM34*, *DMAP1***, IL8	15	0.02	943.7
EVI1-LHXF-HNF1-**NFκB**-ETSF^g^	***ICAM1*, *TOMM34*, *DMAP1***, IL8	15	0.01	1213.3
**NFκB**-EGRF-ETSF-SP1F-ZBPF^g^	***ICAM1*, TXNRD1, YAP1**, ABCG2	15	0.22	70.8
EVI1-HNF1-HOXF-**NFκB**-ETSF^g^	***ICAM1*, *TOMM34*, *DMAP1***, IL8	15	0.02	943.7
EBOX-ZBPF-**NFκB**-MAZF-PAX5^g^	***KCNN4*, YAP1, FADS3**, ABCG2	15	0.13	116.3

Shown are the selected models and their matches in the clusters A and B measured using FrameWorker, and hits in Genomatix promoter database (GPD) measured using ModelInspector of Genomatix Suite 3.4.1 [108]. In general, the distance between two transcription factor binding elements was limited to 5 to 150 base pairs. Genes in bold are orthologs; those in italic and bold contain conserved nuclear factor-κB (NF-κB) or p53 binding sites in orthologous promoter sets; underlined genes contain known NF-κB binding site. ^a^Percentage of matches in p53 or NF-κB group in the cluster for that model. ^b^Percentage of hits in all human promoters (55,207) in GPD for that model. ^c^The ratio between percentage of matches in the cluster and percentage of hits in the entire human promoters of GPD for that model [116]. ^d^Including 17 input genes with predicted p53 binding sites in cluster A. p53 is shown in bold. ^e^Including 26 input genes with predicted NF-κB binding sites in cluster B. NF-κB is shown in bold. ^f^Model searching only covered five transcription factor families: NF-κB, p53, signal transducer and activator of transcription (STAT), activator protein (AP)-1, and early growth response family (EGRF). (-) and (+) indicate strand direction of EGRF binding sites. ^g^This distance was set to 5 to 200 base pairs.

By contrast, the predicted transcription factor models exhibited greater diversity when connecting NF-κB with other transcription factors. The major transcription factors associated with NF-κB were ETSF (human and murine ETS1 factors; 8/14) and ZBPF (zinc binding protein factors; 8/14). In most cases the locations of NF-κB binding sites were near to either ETSF or ZBPF, except in two cases, where NF-κB sites were separated from ETSF or ZBPF by PAX5 or EGRF (early growth response family). We noticed that the selectivity of these models containing five TFBSs was much greater than that of other ones. It is therefore possible that cooperation of ETSF-NF-κB or ZBPF-NF-κB with other transcription factors is part of NF-κB transcriptional regulatory mechanisms. These transcription factor modules are over-represented in the promoter regions of genes with conserved and confirmed NF-κB binding sites, such as *IL8 *(6/14), *ICAM1 *(6/14), and *IL6 *(5/14); genes with conserved NF-κB binding sites, such as *DMAP1 *(8/14), *TOMM34 *(7/14), and *KCNN4 *(5/14); genes containing an orthologous promoter, such as *YAP1 *(6/14); and *ABCG2 *(8/14), which did not belong to any of these categories (Table 3). Furthermore, we identified three NF-κB-EGRF and one NF-κB-STAT models if only five transcription factor families (namely NF-κB, p53, STAT, AP-1, and EGRF) were included in the analysis (Table 3). Both models containing NF-κB with either EGRF or STAT were observed in seven common genes: *ICAM1*, *TOMM34*, *DAMP1*, *FADS3*, *KRT8*, *ABCG2*, and *MLPH*.

### Gene expression and promoter binding activity modulated by doxorubicin or tumor necrosis factor-α

**Figure 6:**
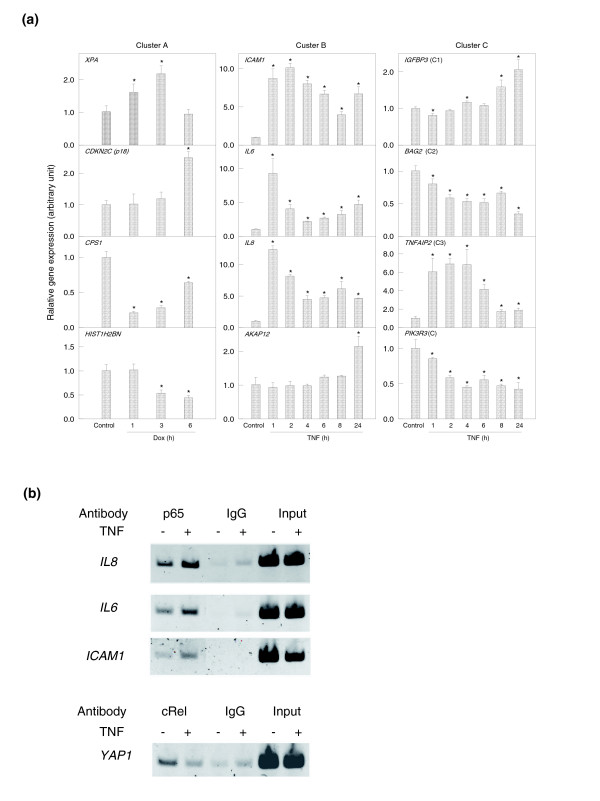
Basal and inducible gene expression, and promoter binding activity were modulated by doxorubicin or TNF-α. **(a) **Human keratinocyte (HKC) cells were treated with doxorubicin (Dox; 0.5 μg/ml, left panels), and the UM-SCC 6 cell line was treated with tumor necrosis factor (TNF)-α (2000 U/ml, center and right panels) for different periods, as indicated. Total RNA was harvested by Trizol and genes selected from clusters A to C were analyzed by real-time reverse transcription polymerase chain reaction (RT-PCR). The data are presented as the mean plus standard deviation from triplicates with normalization by 18S ribosome RNA. '(C1)', '(C2)', and '(C3)' refer to the three subclusters of cluster C. '(C)' refers to genes in cluster C outside subclusters C1 to C3. **(b) **Chromatin immunoprecipitation assays were performed in UM-SCC 11A cells using rabbit polyclonal anti-p65 or cRel antibodies with IgG isotype control.

To obtain experimental evidence on the predicted role of p53 and NF-κB in regulating gene expression of the clusters, we compared expression levels of selected genes at baseline and following treatment with classical inducers for p53 (doxorubicin) or NF-κB activation (tumor necrosis factor [TNF]-α), using real time RT-PCR. For study of cluster A genes, HKCs were used and treated with doxorubicin to ensure functional p53 status, because a variety of deficiencies in p53 expression and function were observed in UMSCC cell lines with different p53 status (Friedman, unpublished data). As shown in the left panels of Figure 6a, doxorubicin treatment either induced or suppressed expression levels of genes from cluster A in a time-dependent manner. For genes from clusters B and C, in which NF-κB promoter binding motifs were prevalent, TNF-α treatment significantly modulated the expression of multiple genes in UM-SCC 6 cells (Figure 6a; middle and right panels). To confirm whether predicted NF-κB binding sites in promoters from cluster B genes are bound by NF-κB components, ChIP binding assay was performed using anti-NF-κB antibodies (p65 and cRel). The results showed the promoter binding activity of *ICAM1*, *IL8*, *IL6*, and *YAP1 *genes from cluster B (Figure 6b). A strong basal and TNF-α induced p65 binding activity in *IL8 *promoter, and a weaker basal and significant TNF-α induced p65 binding activity were observed in *IL6 *and *ICAM1 *promoters, respectively (Figure 6b). Binding of NF-κB family member cRel to the predicted cRel motif on the *YAP1 *promoter was detected and inhibited by TNF-α (Figure 6b). Minimal nonspecific binding activities were observed in the negative controls using isotype IgGs. In addition, we also observed constitutive p53 binding activity to the promoters of *HIST1H2BD *and *HIST1H2BN *from the cluster A gene list (Yang and coworkers, unpublished data).

## Discussion

Gene expression profiles have been intensively studied to identify critical gene expression signatures related to heterogeneity in HNSCC phenotypes [1-8,56]. However, the underlying transcriptional regulatory mechanisms have not been well defined. Until recently, genome-wide analysis of transcriptional regulation involving multiple signal pathways and transcription factors have mostly been conducted in the prokaryotic and lower eukaryotic organisms, such as *Escherichia coli *and yeast. Little information regarding transcriptional control of global gene expression has been generated from large-scale analysis of gene expression profiles in cancer related investigations. Utilizing array technology together with up-to-date bioinformatics analyses and biologic information, we found evidence for increased prevalence and differential distribution of TFBSs for five transcription factors in association with differentially expressed gene signatures in subsets of HNSCC cell lines. The five transcription factors p53, NF-κB, AP-1, STAT3, and EGR-1 have previously been implicated by our laboratory and others [21,22,24,25,30-33,36,41,50-52] as independent factors that contribute to malignant progression of HNSCCs. However, the overall biologic significance and potential scope of the contribution of these transcription factors in regulating global and heterogeneous gene expression have not previously been defined. Our results suggest that over-expressed gene clusters in human HNSCC subgroups identified by global expression profile most likely involve the regulation of transcription factors p53, NF-κB, and AP-1. In addition, the broad repertoire of genes over-expressed by most HNSCCs may be co-regulated by other transcription factors such as STAT3 and EGR1. Enrichment for binding sites of NF-κB, AP-1, STAT3, or EGR1 was found in the promoters of genes in cluster B that are involved in cell survival, inflammation, and angiogenesis (Figure 2 and Table 2). Some of the genes (*IL6*, *IL8*, *YAP1*, and *BIRC2*) were associated with SCC metastatic progression and aggressive phenotypes in previous studies [9,38], suggesting that these transcription factors may cooperate to activate gene signatures that are important in pathogenesis and increase the malignant potential of HNSCCs with a wild-type p53-like expression pattern.

A remarkable observation from this study is the apparent inverse relationship between expression of cluster A and B genes and their association with dominant prevalence of binding sites of p53 in cluster A genes, and NF-κB, AP-1, and other transcription factors in cluster B genes. Cluster A genes were expressed at higher levels in most cells with mutant p53 and exhibited a higher frequency of p53 and lower frequency of AP-1 binding sites than those present in promoters of genes in other clusters or vertebrate promoters (Figure 3). The segregation of HNSCC cells into subsets with these gene expression clusters revealed a relationship with p53 mutation status in most of the cells, which supports the importance of predicted p53 binding sites (Figure 3). In cluster A genes the increased prevalence of predicted p53 binding motifs (Figure 3 and Table 2) were consistent with experimental data indicating that selected genes from the list could be modulated by doxorubicin treatment in HKCs that have normal p53 status (Figure 6a). Surprisingly, however, cluster A genes were over-expressed by UM-SCC cells with mutant p53 genotype, and not by cells with wild-type p53-like expression pattern (Figure 3). A detailed mechanistic study of the role played by mutant p53 in cluster A gene expression is underway. Conversely, cluster B genes with promoters containing known or putative motifs for NF-κB were highly represented in HNSCCs with a wild-type p53-like expression pattern. This suggests that p53 status may also affect expression of NF-κB regulated genes. One possible explanation could be related to a mechanism proposed by Perkins and colleagues [57-59], who previously showed that lack of functional p53 or p14^ARF ^can permit activation of NF-κB, whereas p14^ARF ^or p53 activation can result in a repression of NF-κB regulated genes through competition for co-factor CBP/p300 or phosphorylation of p65 thr 505. Studies of the mechanism(s) underlying this apparent inverse relationship between p53 and NF-κB regulated genes in HNSCC cells are in progress (Friedman, unpublished data).

In this study, we observed segregation of nine out of 10 of the UM-SCC lines by *p53 *genotype, with the exception of UM-SCC 11B cells (Figures 1 and 2). The UM-SCC 11B cell line, which exhibited a p53 mutation by sequence analysis and heterogeneous p53 protein expression in different subpopulations by immunohistochemistry (Figure 1b), clustered with its parental UM-SCC 11A and other UM-SCC cell lines that exhibit low expression of wild-type p53 (Figures 1 and 2). The low mRNA and protein expression of wild-type p53 with deficient p53 function were previously reported in breast cancer cell lines and tissue specimens [60,61]. At this stage, we have not yet determined whether the p53 related subgrouping of UM-SCC cells by gene profiling could result from loss of wild-type p53 function in the UM-SCC cells with wild-type p53-like expression pattern, differences in function of the various *p53 *mutants, or activation or interactions with other p53 family members or co-factors. Preliminary data revealed the presence of low transducing activity by reporter gene assay in UM-SCC 11B and other UM-SCC cells with a wild-type p53-like expression pattern, which is in contrast to the greater activity observed in some UM-SCC cells with gain-of-function mutant p53 (Friedman, unpublished data). In addition, multiple p53, p63, and p73 family members can compete for the same binding motifs and differentially regulate various p53 family gene programs, such as those recently implicated in survival and adhesion of HNSCCs [62]. Exceptions observed in our study may be useful in dissecting whether certain p53 mutations or alternative family members affect overall gene expression profiles. Determination of the regulation of these genes will require further study of the potentially complex contribution of expression, phosphorylation, and co-factor interaction of wild-type and p53 mutants, and possibly other p53 family members such as p63 and p73.

In cluster A, about 25% of genes are involved in chromosome structure and functions by GO annotation (Additional data file 2), including eight genes in histone H2A and H2B families. However, the biologic significance of heterogeneous expression of the H2 histone gene family in cancer biology and the relationship to p53 regulatory mechanisms are not well understood. It is known that the core structure of the nucleosome is dependent upon both histone-histone and histone-DNA interactions, and alterations of chromatin structures and modifications of histone by acetylation and phosphorylation are basic processes for activation or repression of gene expression [63]. For example, in H2AX-deficient mice, embryonic stem cells exhibited impaired recruitment of specific DNA repair complexes to ionizing radiation-induced nuclear foci [64]. Histone H2B has been suggested to play a specific role in UV-induced DNA repair processes in yeast [65], whereas histone H2B-elicited ubiquitylation may play a role in gene activation and affect gene silencing [66]. Mdm*2 *is a negative regulator of p53 that can interact directly with histones and induce monoubiquitylation of histones H2A and H2B [67,68]. Yu and coworkers [68] observed that rapid p53-mediated inhibition of cell cycle progression was induced by DNA-damaging agents in differentiated neuroblastoma cells, resulting in induction of cdk2-cyclin E expression followed by phosphorylation of histone H2B and cell death. In addition, p53 is also able to recruit p300 to the p21 promoter that leads to targeted acetylation of chromatin-assembled core histones H2A, H2B, H3, and H4 [69]. The possible effects of differential expression of the histone genes in transcription or DNA integrity in HNSCCs over-expressing mutant p53 warrant further investigation.

Cluster B genes were over-expressed by UM-SCC cells with wild-type p53-like expression pattern (Figure 2). Functional categories of about 40% genes in cluster B with dominant NF-κB binding sites belong to cell proliferation and signal transduction (Table 2). TNF-α induced rapidly and significantly increased expression of several known NF-κB targeted genes, including *ICAM1*, *IL6*, *IL8 *and *TNFAIP2*, but the mechanisms are less well understood for the rest of the genes presented (Figure 6a). We previously showed that, in HNSCCs, NF-κB elicited over-expression of cytokine and growth factor genes that promote inflammation and angiogenesis, such as *IL1*, *IL6*, *IL8*, *GRO-1*, *GM-CSF*, and *VEGF *[21,22,24,25,37,49,70]. Molecular profiling of transformed and metastatic murine SCCs showed that *Gro1 *(*IL8 *homolog), antiapoptotic gene *cIAP-1 *(*cIap-1*/*Birc2*), and *Yap65 *(*YAP 1*) were upregulated and clustered together in association with activation of NF-κB and tumor growth, metastatic progression, and angiogenesis [37,38]. Chung and coworkers [71] recently identified human homologs of a set of 99 genes from our murine tumor model. These genes are modulated by NF-κB and highly represented in a gene cluster and subset of patients with HNSCC who have poor prognosis [71]. In addition, some cluster B genes, such as *BIRC2 *(*cIAP-1*), *YAP1*, and *KRT18 *were also identified by another laboratory using 5 UM-SCC cell lines resistant to cisplatin [5]. Jeon and coworkers [1] studied 25 UM-SCC lines, including 11A, 11B, 22A and 22B; three of the genes in their upregulated gene cluster, namely *IL8*, *KRT8*, and *TXNRD1*, overlapped with our cluster B gene list. Recently, Roepman and coworkers [9] reported that cluster B genes *IL6*, *IL8*, *YAP1*, and *BIRC2 *are upregulated in metastatic HNSCC tumor specimens. *ICAM1 *is another NF-κB regulated gene in the cluster B list and is implicated in adhesion in a wide range of inflammatory and immune responses, and carcinogenesis [72-75]. Together, these data indicate that heterogeneity in expression of genes in clusters B and C are important in the malignant phenotype and therapeutic resistance, and that NF-κB and other transcription factors contribute to their differential expression in HNSCCs.

Increased prevalence of STAT3 and EGR1 binding motifs were predicted in the promoters and distributed throughout the gene clusters over-expressed by UM-SCC cells, when compared with those in vertebrate promoters, indicating that these transcription factors could play a broader or co-regulatory role with other transcription factors in gene expression in HNSCC tumorigenesis [31-33,36]. In addition, the occurrence of three EGRF-NF-κB and one STAT-NF-κB putative modules were identified, which is consistent with previous examples indicating that NF-κB can actively interact with STAT3, EGR1, and other transcription factors to regulate cytokine and growth factor expression, and receptor kinase activation [76-79]. Our previous experimental data are also consistent with the hypothesis that NF-κB may interact with EGR1, activated by hepatocyte growth factor (HGF) and the tyrosine kinase receptor c-MET, to co-regulate gene expression in HNSCC [36,48]. EGR1 can be activated through HGF/c-Met tyrosine kinase receptor and protein kinase C dependent mechanisms and induce *PDGF *and *VEGF *[48]. HGF promoted expression of angiogenesis factors in tumor cells through both mitogen-activated protein kinase kinase and phosphatidylinositol 3-kinase dependent pathways [48]. Over-expression of *c-Met *expression enhanced the expression of angiogenesis factors *IL8*, *VEGF*, and *PDGF *in response to *HGF in vitro *[48], and increased tumorigenesis and metastasis in the tumor microenvironment [80].

Identification of TFBSs by sequence similarity only indicates the potential for physical binding of transcription factors to their corresponding regulatory regions. These binding motifs do not all necessarily play functional roles in the biologic context. Determination of conservation of *cis*-regulatory elements across species, or so-called phylogenetic footprinting [81,82], is useful in predicting a functional role of *cis-*regulatory motifs in transcriptional regulation [10,82]. We analyzed conserved TFBSs by two steps at the sequence level, including annotation of orthologous genes and study of the similarity of TFBSs in aligned regulatory regions. We found a majority of the putative TFBSs (more than about 60%) appeared in orthologous promoter sets of cluster A or cluster B (Table 2), and conserved binding sites for p53 and NF-κB were observed in five and ten genes of the two clusters (Figure 4). These data suggest that these predicted TFBSs are likely to be functional in evolution, and the activities of some predicted TFBSs have been tested experimentally. As shown in Figure 6a, among the genes we selected for experiment, ten out of 12 gene promoters fell within orthologous regions (except *HIST1H2BN *and *IL8*; Table 2). Their levels of gene expression were regulated by doxorubicin or TNF-α (Figure 6a).

It is widely believed that transcriptional regulation of gene expression is most often accomplished by functional cooperation of multiple transcription factors rather than a single factor. In the promoters of cluster A genes, p53 binding motifs were predicted to co-localize with TBPF motifs for TATA-binding proteins (Table 3). As a regulatory module, p53-TBPF interacts with other transcription factors that could contribute significantly to the controlling mechanism of cluster A gene expression regulating chromosome structure, function, and stability (Table 3). In addition, a putative common regulatory module for all eight histone genes in cluster A was identified as TBPF-ECAT-PCAT with high selectivity (Figure 5). It is interesting that the predicted p53 binding sites in four histone genes are close to this module. Using BiblioSphere PathwayEdition of Genomatix, the functional co-citations of p53-NFYC (nuclear factor YC) and p53-YB-1 (Y-box-binding protein 1) were observed, and NFYC and YB-1 belong to ECAT and PCAT families. It has been shown that YB-1 (also known as DNA binding protein B) accumulates in the nucleus under genotoxic stress only when cells retain wild-type p53, whereupon YB-1 inhibits p53 activity [83]. The NFY family, including A, B and C subunits, are histone-like CCAAT-binding trimers and are specifically required for consensus CCAAT-box binding in enhancer regions [84-86]. The core regions of NFYC and NFYB proteins exhibited high sequence similarity with histones H2A and H2B, respectively [84,87,88], and NFYC has been shown to be an important target of p53 regulation in wild-type p53 mediated gene repression [89,90].

In addition to the dominant NF-κB binding motifs on the promoter regions of cluster B, we also captured 14 candidate NF-κB related regulatory models linked with orthologous promoter sets, or the conserved or experimentally defined NF-κB binding sites (Table 3). Six genes, namely *IL6*, *IL8*, *ICAM1*, *KCNN4*, *TOMM34*, and *DMAP1*, which contain phylogenetic conserved or known NF-κB binding sites were highly enriched in these selected models. The majority of the regulatory modules contain ETSF-NF-κB, ZBPF-NF-κB and EGRF-NF-κB with other TFBSs. The ETS family is a large set of transcription factors that is characterized by an evolutionally conserved ETS domain; they are related to cancer cell growth, signal transduction, angiogenesis, cell proliferation, and apoptosis [91-94]. ZBP-89 (also called ZNF-148), a main ZBPF member, is a Krüppel-type zinc-finger protein and is involved in transcriptional regulation of a variety of genes [95], cell growth arrest [96,97], and apoptosis and cell death [96]. Borghaei and coworkers [98] found that both ZBP-89 and NF-κB binding to SIRE (stromelysin IL-1 responsive element) sites of the matrix metalloproteinase-3 promoter in response to inflammatory cytokines [98]. Coordinated binding of ZBP-89, SP1, and NF-κB p65/p50 in the *ENA-78 *(epithelial neutrophil-activating peptide-78) promoter play a major role in the regulation of *ENA-78 *expression in Caco-2 human colonic epithelial cells [99]. Thus, the regulation of genes in cluster B by NF-κB is most likely linked to ETS members or ZBPF. We are currently conducting studies to confirm this co-operation of transcription factors and their binding to the promoter sequence experimentally.

In our ten UM-SCC cell lines, unique and novel gene expression signatures were identified by cDNA microarray analysis, and the newly discovered regulatory mechanisms that control such gene expression signatures (predominantly involving transcription factors) were revealed. Important genes within the expression profiles, and the related transcriptional regulatory mechanisms identified, are consistent with observations from clinical studies in more complex tissue specimens in independent studies at our and other institutions, supporting the biologic and clinical relevance of genes within clusters identified in our study in an experimental cell system [5-8,13,16-20,31,32,41,70,71].

## Conclusion

A distinct molecular gene classification of HNSCC cells was obtained with differentially expressed gene clusters associated with different p53 genotype and protein expression status. The prevalences of p53, NF-κB, and AP-1 binding sites were observed in the promoter regions of different gene clusters, suggesting their importance for the subgrouping of HNSCCs. Increases in STAT3 and EGR1 binding sites were observed in all over-expressed gene clusters compared with vertebrate promoters, indicating their broader role in co-regulating the expression of genes in HNSCCs. The importance of these transcriptional regulatory elements in clustered gene expression by UM-SCC cells was supported by the prediction of several regulatory modules in different gene clusters, experimentally modulated gene expression under doxorubicin and TNF-α, and NF-κB binding activity measured by ChIP assays. Therefore, our finding of the predominant role of p53, NF-κB, and other transcription factors provides a useful clue for elucidating the mechanisms that control differential expression of gene clusters and related molecular classifications in HNSCCs, and to develop targeted therapeutics in the future.

## Materials and methods

### Cell lines

Ten established HNSCC cell lines, namely UM-SCC 1, 5, 6, 9, 11A, 11B, 22A, 22B, 38, and 46, were obtained from the University of Michigan series of HNSCC cell lines (Ann Arbor, MI, USA), as described previously and in Table 1[26,48,70]. HKCs were obtained from four individuals (Cascade Biologics Inc., Portland, OR, USA), and cultured following the manufacturer's suggestions.

### DNA sequencing

Genomic DNA was isolated using Trizol method, in accordance with the manufacturer's suggestions (Invitrogen, Carlsbad, CA, USA). DNA sequencing was conducted using PCR with reported primers for *p53 *exons 4 to 9, and samples were sequenced bidirectionally using Applied Biosystems DNA sequencers (Applied Biosystems, Foster City, CA, USA), in accordance with the manufacturer's protocol, at the NIDCD (National Institute on Deafness and Other Communication Disorders) Core Sequencing Facility. All *p53 *mutations were confirmed in independent PCR reactions to ensure that the mutation was not an artifact of PCR.

### Immunocytochemistry

Immunostaining of p53 protein was performed in cultured UM-SCC cells. Briefly, UM-SCC cells (1 to 2 × 10^4^) were plated on eight-well chamber slides (Lab-Tek, Naperville, IL, USA) for 2 to 3 days, fixed, and permeabilized in freshly made cold methanol:acetone (1:1). Then, 1% hydrogen peroxide (Fisher Scientific, Fair Lawn, NJ, USA) was used for blockade of endogenous peroxidase. Ten per cent blocking serum (Vector Labs, Inc., Burlingame, CA, USA) was used to block the nonspecific binding sites for 20 min and removed without washing. The samples were incubated with the primary antibodies, namely 2 μg/ml mouse anti-human p53 (DO-1, IgG_2a_; Calbiochem, EMD Biosciences, San Diego, CA, USA) or isotype control mouse IgG (Cat# I2000, Vector Labs, Inc.), which were diluted in phosphate-buffered saline with 10% blocking serum overnight at 4°C. The samples were blocked with 5% serum for 20 min and incubated with the secondary biotinylated antibody for 30 min, followed by 30 min incubation with biotin/avidin horseradish peroxidase conjugates (Vectastain Elite ABC kit; Vector Labs, Inc.) and chromogen DAB (ciaminobenzidine tetrahydro-chloride; Vector Labs, Inc.), in accordanc with the manufacturer's specifications.

### Microarray experiments and data collection

The experimental methods used for microarray were recently described [44,100]. Briefly, the cDNA microarray chips of 24,000 human elements were developed and printed by National Human Genome Research Institute (Bethesda, MD, USA). Total RNA was isolated from UM-SCC cell lines or HKCs, reverse transcribed, labeled with Cy5, and combined with the Cy3 labeled cDNA reverse transcribed from human universal reference RNA (Stratagene, La Jolla, CA, USA). The labeled targets were hybridized on to the cDNA array chip at 65°C overnight. Fluorescence intensity was obtained using a GenePix 4000 microarray scanner with GenePix Pro software (Axon Instruments, Union City, CA, USA).

### Microarray data analysis according to the p53 related expression pattern of UM-SCC cell lines

The 24,000 cDNA microarray chips contained a total of 23,220 spots, which includes known genes, expressed sequence tags, hypothetical proteins, and control spots. There were 12,270 known gene sports on the arrays [44], where a list of 9,273 known genes with good quality were generated and subjected to principal components analysis and genome-wide gene expression hierarchical clustering. The results segregated cells into three distinct groups, HKCs, UM-SCC cells with wild-type p53-like expression pattern (including UM-SCC 11B), and most cells with mutant p53 [44]. We have tested the subgrouping of UM-SCC cells using four strategies: principle components analysis of 9,273 known genes; hierarchical clustering analysis of 9,273 known genes; hierarchical clustering analysis of genes that exhibited at least twofold changes in gene expression among keratinocytes, five UM-SCC lines with mutant p53, and five UM-SCC lines with wild-type p53-like status (including UM-SCC 11B cells); and hierarchical clustering analysis of the genes with at least twofold changes in gene expression among the groups when UM-SCC 11B was included in the mutant p53 group. Under all four circumstances, UM-SCC 11B line is always grouped with UM-SCC cells with wild-type p53-like group, but not with the group of cells with mutant p53. The significance of this grouping of cells has been tested by multiple statistical methods in the 'class prediction analysis' provided in BRB-Array Tools developed by the US National Institutes of Health, in which UM-SCC 11B was predicted to belong to the group designated wild-type p53-like (*P *< 0.001) [45]. Based on these observations, we defined the subset of UM-SCC cells with wild-type p53 genotype plus UM-SCC 11B cells as exhibiting a wild-type p53-like expression pattern.

In order to analyze further the differential gene expression between HKCs and UM-SCC cells with different p53 status, we selected genes that satisfy at least one of the following criteria (two-tailed *t*-test, *P *< 0.05): twofold or greater change in average gene expression between UM-SCC cells in either group when compared with the average gene expression by HKCs; and twofold or greater change in average gene expression between ten tumor cell lines and HKCs. Hierarchical clustering was performed using Stanford University software Cluster 2.11 [101]. Missing data from microarray were estimated using K imputer of SAM 2.11 [102]. Normalized two-based log ratios for 1,011 genes were median-centered within each gene and array for all of the cluster analyses. The clustering results were obtained based on average linkage with one minus Pearson correlation distance metrics. The expression of clustered genes was visualized using Java Treeview [103].

A mixed model based F test was used to analyze differential gene expression among three groups: HKCs, UM-SCC cell lines with wild-type p53-like expression patterns, and UM-SCC cell lines with mutant p53 expression patterns. Such a model was used to test the fixed effects of random factors [104,105]. Each gene expression level was measured independently for each subject with homogeneous variance. The normalized model for this study is as follows:

Y_ijg _= μ + S_j _+ G_g _+ Ex_i(c,w,m) _+ ε_ijg_

Where Y_ijg _denotes response of gene g for subject j in group i (c, w, m); μ is a fixed effect that represents an overall mean value; S_j _is the random effect of each subject; G_g _is the random effect of each gene; ε_ijg _is the random error; Ex_(c,w,m) _is the fixed effect for different gene expression type; c is HKCs; w is wild-type p53-like; and m is mutant p53. Restricted maximum likelihood was used to estimate the covariance parameters in the model. The F test was performed in mixed model using SAS 9.1 program (SAS Institute Inc. Cary, NC, USA). *P *< 0.05 between two groups was considered statistically significant.

### Gene Ontology annotation

GO annotation was performed using Onto-Express [46], AmiGo [106], and Entrez Gene database of National Center for Biotechnology Information (National Institutes of Health, Bethesda, MD, USA) [107]. Onto-Express dynamically calculates *P *values for the GO term based on the abstraction level chosen by the user. A hypergeometric distribution was used to calculate the significance values in the probability model [46]. Corrected *P *< 0.05 was used as cutoff value in this study.

### Prediction of transcription factor binding sites on promoters of clustered genes

Promoter sequences of selected genes were extracted using ElDorado task of commercial Genomatix software suite 3.4.1 [108]. The promoter regions were defined as 'optimized' regions by Genomatix Suite 3.4.1, in which the average 'optimized' length of 159,207 vertebrate promoters is 632 bp, usually including about 500 bp upstream of the most 5' mapped transcription start site and about 100 bp downstream from the most 3' mapped transcription start site. The vertebrate promoters in GPD consist of 55,207 from human, 69,108 from mouse, and 35,190 from rat. GEMS Launcher of Genomatix is an integrated software package and contains multiple tasks of sequence analysis and functional genomics, including MatInspector. MatInspector is used to search transcription factor matrix matches based on position weight matrices, which provide powerful estimates for searching for TFBSs and have been used successfully to detect functional transcription factor elements [109-111]. To reduce false-positive findings, MatInspector also used optimized transcription factor matrix thresholds and transcription factor family concept, and incorporated data from independent publications [112,113].

In this study, the Gene2Promoter task in Genomatix was used to analyze a set of genes (cluster) when searching for TFBSs through MatInspector. All matrices of p53, NF-κB, and AP-1 families were used. The matrices for p53 family included the full length, 5' half site, or 3' half site of its binding motifs. NF-κB family included five subunits: RelA (p65), RelB, cRel, NF-κB1 (p50), and NF-κB2. AP-1 family consisted of c-Jun, JunB, JunD, c-Fos, FosB, Fra-1, Fra-2, BATF, JDP2, and SNFT. We chose matrix STAT3.01 for the STAT3 search, and matrices EGR1.01 and EGR1.02 for the EGR1 search. Default matrix indices (core similarity: 0.75; matrix similarity: optimized) were set during TFBS searching.

The *P *value of TFBS frequency in a given cluster was calculated by MatInspector. It refers to the probability of obtaining an equal or greater number of sequences with a match in a randomly drawn sample of the same size as input sequence set [108]. *P *calculation was based on 'positional bias' [114] and the precalculated number of promoter matches in GPD. The lower this probability, the greater is the importance of the observed transcription factor. The χ^2 ^test for TFBSs distributed in different clusters was also conducted.

### Prediction of orthologous promoter regions and regulatory modules of multiple transcription factor binding sites

To find conserved TFBSs, we extracted orthologous promoter sets in proximal promoter region with average 'optimized' length among human, mouse, and rat for every gene of interest using 'Comparative genomics' in ElDorado. Multiple sequence alignment of each same promoter set was carried out using the DiAlignTF task of GEMS Launcher. DiAlignTF is able to reduce false-positive findings and display the most likely functional TFBSs within orthologous promoters [112].

Another task, FrameWorker, offers a technology with which to define a common framework of TFBSs from a set of promoter sequences, which was used to construct promoter models consisting of at least two TFBSs for each clustered gene. Such models represent the smallest functional transcriptional unit and putative regulatory module [115]. In this study, we used two sets of genes with either p53 or NF-κB binding sites. In general, the distance of two TFBSs was limited to 5 to 150 bp. For the group with NF-κB binding sites, the distance was set to 5 to 200 bp in order to search models containing four or five TFBSs. Default values were used for other parameters. Such frameworks were refined further with comparative genomics. However, in the p53 group, all genes in the putative models contain at least one p53 binding site. In each model, there was at least one orthologous promoter contains conserved p53 binding sites. The minimal number of the selected genes was seven for two TFBSs, five for three TFBSs, and four for four TFBSs. In the group containing NF-κB binding sites, only models satisfying the following conditions were selected: they contained at least one NF-κB binding site; at least 60% matched gene promoters contained orthologous regions among human, mouse, and rat. The minimal numbers of selected genes were ten for two TFBSs, six for three TFBSs, five for four TFBSs, and four for five TFBSs. Considering the large distance used in defining models containing four to five TFBSs, we set an additional condition; specifically, the model had to contain at least a gene with conserved or known NF-κB binding sites.

The predicted models were then used to scan the entire human database of GPD, containing 55,207 promoters, using ModelInspector of GEMS Launcher. The number of hits and percentage of hits in GPD were traced. The percentage was also used to calculate selectivity in order to evaluate the putative models. 'Selectivity' was calculated using the following formula: percentage of matches in the cluster (the third column in the Table 3) divided by percentage of hits in GPD (the fourth column in the Table 3) [116]. The former refers to proportion of matches in certain gene clusters.

### Basal and inducible gene expression in HKCs and UM-SCC cells

Gene expression profiles generated from microarray were confirmed by real-time quantitative RT-PCR using the Assays-on-Demand™ Gene Expression Assay (Applied Biosystems, Foster City, CA, USA). Briefly, cultured UM-SCC cells at 70% to 80% confluence were treated with or without TNF (2,000 units/ml, Knoll Pharmaceutical Company, Whippany, NJ, USA) or doxorubicin (0.5 μg/ml, Sigma, St. Louis, MO, USA) for different time points, and total RNA was isolated using Trizol (Invitrogen, Carlsbad, CA, USA). cDNA synthesis was performed by using High-Capacity cDNA Archive Kit (Applied Biosystems) to synthesize single-stranded cDNA from total RNA samples, in accordance with the manufacturer's protocol. The PCR cycling was carried out by adding 30 ng cDNA in 30 μl of PCR reaction mix (1.5 μl of 20× assay mix and 15 μl of 2× TaqMan Universal Master Mix; Applied Biosystems). Amplification conditions were as follows: activation of enzymes for 2 min at 50°C and 10 min at 95°C, followed by 40 cycles at 15 s at 95°C and 1 min at 60°C. Thermal cycling and fluorescence detection was done using an ABI Prism 7700 Sequence Detection System (Applied Biosystems). Relative quantitation of the expression was calculated by normalizing the target gene signals with the 18S endogenous control. An arbitrary unit was calculated after setting C_T _equal to 40 as undetectable expression level.

### Chromatin immunoprecipitation assay

ChIP assays were performed using the EZ ChIP assay kit (Upstate Biotechnology, Waltham, MA, USA), following the manufacturer's directions. Briefly, cultured UM-SCC cells at 70% to 80% confluence were treated with or without TNF-α (2,000 unit/ml) for 1 hour. DNA and proteins were cross-linked by 1% formaldehyde, whole cell lysates then were harvested, and sonicated using SONICATOR XL2020 (Misonix Inc, Farmingdale, NY, USA). Pre-cleared chromatin was immunoprecipitated with a rabbit polyclonal antibody against p65 (Upstate Biotechnology), or c-Rel subunits (Santa Cruz, Biotechnology, Inc, Santa Cruz, CA, USA) of human NF-κB or normal rabbit IgG (Upstate Biotechnology), followed by incubation with protein A-agarose saturated salmon sperm DNA (Upstate Biotechnology). Precipitated DNA was analyzed by PCR (35 cycles) with Platinum Taq DNA Polymerase (Invitrogen, Carlsbad, CA). The PCR products were analyzed on a 2% agarose E-Gel (Invitrogen). Primers for the *IL8 *promoter (-121 to +61 bp) were 5'-GGGCCATCAGTTGCAAATC-3' (forward) and 5'-TTCCTTCCGGTGGTTTCTTC-3' (reverse). For the *IL6 *promoter (-203 to -60 bp) they were 5'-TGCACTTTTCCCCCTAGTTG-3' (forward) and 5'-TCATGGGAAAATCCCACATT-3' (reverse). For the *ICAM1 *promoter (-385 to -157 bp) they were 5'-GCAGCCTGGAGTCTCAGTTT-3' (forward) and 5'-TCCGGAATTTCCAAGCTAAA-3' (reverse). Finally, for the *YAP1 *(Yes-associated protein 1) promoter (-309 to -164) they were 5'-TAGCAACTTGCAGCGAAAAG-3' (forward) and 5'-GCCTCAAACGCCAAAACTAA-3' (reverse). All of the above PCR amplified regions contained at least a putative NF-κB binding site, as predicted by Genomatix Suite 3.4.1.

## Additional data files

The following additional data are available with the online version of this paper. Additional data file 1 shows list of genes in cluster C (over-expressed in UM-SCC cells). Additional data file 2 includes GO annotations of genes in clusters A to C (over-expressed in UM-SCC cells) and of genes in clusters D to F (under-expressed in UM-SCC cells). Additional data 3 provides sequences, location, and matrix similarity of putative TFBSs in genes in clusters A and B (over-expressed in UM-SCC cells).

## Supplementary Material

Additional data file 1 Shown is a list of genes in cluster C (over-expressed in UM-SCC cells). Click here for file

Additional data file 2 Included are GO annotations of genes in clusters A to C (over-expressed in UM-SCC cells) and of genes in clusters D to F (under-expressed in UM-SCC cells). Click here for file

Additional data file 3 Provided are sequences, location, and matrix similarity of putative TFBSs in genes in clusters A and B (over-expressed in UM-SCC cells). Click here for file

## References

[B1] Jeon GA, Lee JS, Patel V, Gutkind JS, Thorgeirsson SS, Kim EC, Chu IS, Amornphimoltham P, Park MH (2004). Global gene expression profiles of human head and neck squamous carcinoma cell lines.. Int J Cancer.

[B2] Warner GC, Reis PP, Makitie AA, Sukhai MA, Arora S, Jurisica I, Wells RA, Gullane P, Irish J, Kamel-Reid S (2004). Current applications of microarrays in head and neck cancer research.. Laryngoscope.

[B3] Choi P, Chen C (2005). Genetic expression profiles and biologic pathway alterations in head and neck squamous cell carcinoma.. Cancer.

[B4] Gonzalez HE, Gujrati M, Frederick M, Henderson Y, Arumugam J, Spring PW, Mitsudo K, Kim HW, Clayman GL (2003). Identification of 9 genes differentially expressed in head and neck squamous cell carcinoma.. Arch Otolaryngol Head Neck Surg.

[B5] Akervall J, Guo X, Qian CN, Schoumans J, Leeser B, Kort E, Cole A, Resau J, Bradford C, Carey T (2004). Genetic and expression profiles of squamous cell carcinoma of the head and neck correlate with cisplatin sensitivity and resistance in cell lines and patients.. Clin Cancer Res.

[B6] Ginos MA, Page GP, Michalowicz BS, Patel KJ, Volker SE, Pambuccian SE, Ondrey FG, Adams GL, Gaffney PM (2004). Identification of a gene expression signature associated with recurrent disease in squamous cell carcinoma of the head and neck.. Cancer Res.

[B7] Chung CH, Parker JS, Karaca G, Wu J, Funkhouser WK, Moore D, Butterfoss D, Xiang D, Zanation A, Yin X (2004). Molecular classification of head and neck squamous cell carcinomas using patterns of gene expression.. Cancer Cell.

[B8] Hunter KD, Thurlow JK, Fleming J, Drake PJ, Vass JK, Kalna G, Higham DJ, Herzyk P, Macdonald DG, Parkinson EK (2006). Divergent routes to oral cancer.. Cancer Res.

[B9] Roepman P, Kemmeren P, Wessels LF, Slootweg PJ, Holstege FC (2006). Multiple robust signatures for detecting lymph node metastasis in head and neck cancer.. Cancer Res.

[B10] Fickett JW, Wasserman WW (2000). Discovery and modeling of transcriptional regulatory regions.. Curr Opin Biotechnol.

[B11] Sionov RV, Haupt Y (1999). The cellular response to p53: the decision between life and death.. Oncogene.

[B12] Heinrichs S, Deppert W (2003). Apoptosis or growth arrest: modulation of the cellular response to p53 by proliferative signals.. Oncogene.

[B13] Hollstein M, Sidransky D, Vogelstein B, Harris CC (1991). p53 mutations in human cancers.. Science.

[B14] Olivier M, Eeles R, Hollstein M, Khan MA, Harris CC, Hainaut P (2002). The IARC TP53 database: new online mutation analysis and recommendations to users.. Hum Mutat.

[B15] Vousden KH, Lu X (2002). Live or let die: the cell's response to p53.. Nat Rev Cancer.

[B16] Brennan JA, Boyle JO, Koch WM, Goodman SN, Hruban RH, Eby YJ, Couch MJ, Forastiere AA, Sidransky D (1995). Association between cigarette smoking and mutation of the p53 gene in squamous-cell carcinoma of the head and neck.. N Engl J Med.

[B17] Melhem MF, Law JC, el-Ashmawy L, Johnson JT, Landreneau RJ, Srivastava S, Whiteside TL (1995). Assessment of sensitivity and specificity of immunohistochemical staining of p53 in lung and head and neck cancers.. Am J Pathol.

[B18] Nylander K, Schildt EB, Eriksson M, Magnusson A, Mehle C, Roos G (1996). A non-random deletion in the p53 gene in oral squamous cell carcinoma.. Br J Cancer.

[B19] Quon H, Liu FF, Cummings BJ (2001). Potential molecular prognostic markers in head and neck squamous cell carcinomas.. Head Neck.

[B20] Blons H, Laurent-Puig P (2003). TP53 and head and neck neoplasms.. Hum Mutat.

[B21] Ondrey FG, Dong G, Sunwoo J, Chen Z, Wolf JS, Crowl-Bancroft CV, Mukaida N, Van Waes C (1999). Constitutive activation of transcription factors NF-(kappa)B, AP-1, and NF-IL6 in human head and neck squamous cell carcinoma cell lines that express pro-inflammatory and pro-angiogenic cytokines.. Mol Carcinog.

[B22] Duffey DC, Chen Z, Dong G, Ondrey FG, Wolf JS, Brown K, Siebenlist U, Van Waes C (1999). Expression of a dominant-negative mutant inhibitor-kappaBalpha of nuclear factor-kappaB in human head and neck squamous cell carcinoma inhibits survival, proinflammatory cytokine expression, and tumor growth in vivo.. Cancer Res.

[B23] Duan J, Friedman J, Nottingham L, Chen Z, Ara G, Van Waes C (2007). Nuclear factor-kappaB p65 small interfering RNA or proteasome inhibitor bortezomib sensitizes head and neck squamous cell carcinomas to classic histone deacetylase inhibitors and novel histone deacetylase inhibitor PXD101.. Mol Cancer Ther.

[B24] Wolf JS, Chen Z, Dong G, Sunwoo JB, Bancroft CC, Capo DE, Yeh NT, Mukaida N, Van Waes C (2001). IL (interleukin)-1alpha promotes nuclear factor-kappaB and AP-1-induced IL-8 expression, cell survival, and proliferation in head and neck squamous cell carcinomas.. Clin Cancer Res.

[B25] Bancroft CC, Chen Z, Yeh J, Sunwoo JB, Yeh NT, Jackson S, Jackson C, Van Waes C (2002). Effects of pharmacologic antagonists of epidermal growth factor receptor, PI3K and MEK signal kinases on NF-kappaB and AP-1 activation and IL-8 and VEGF expression in human head and neck squamous cell carcinoma lines.. Int J Cancer.

[B26] Yu M, Yeh J, Van Waes C (2006). Protein kinase casein kinase 2 mediates inhibitor-kappaB kinase and aberrant nuclear factor-kappaB activation by serum factor(s) in head and neck squamous carcinoma cells.. Cancer Res.

[B27] Pahl HL (1999). Activators and target genes of Rel/NF-kappaB transcription factors.. Oncogene.

[B28] Richmond A (2002). Nf-kappa B, chemokine gene transcription and tumour growth.. Nat Rev Immunol.

[B29] Li JJ, Westergaard C, Ghosh P, Colburn NH (1997). Inhibitors of both nuclear factor-kappaB and activator protein-1 activation block the neoplastic transformation response.. Cancer Res.

[B30] Hong SH, Ondrey FG, Avis IM, Chen Z, Loukinova E, Cavanaugh PF, Van Waes C, Mulshine JL (2000). Cyclooxygenase regulates human oropharyngeal carcinomas via the proinflammatory cytokine IL-6: a general role for inflammation?. Faseb J.

[B31] Grandis JR, Drenning SD, Zeng Q, Watkins SC, Melhem MF, Endo S, Johnson DE, Huang L, He Y, Kim JD (2000). Constitutive activation of Stat3 signaling abrogates apoptosis in squamous cell carcinogenesis in vivo.. Proc Natl Acad Sci USA.

[B32] Song JI, Grandis JR (2000). STAT signaling in head and neck cancer.. Oncogene.

[B33] Lee TL, Yeh J, Van Waes C, Chen Z (2006). Epigenetic modification of SOCS-1 differentially regulates STAT3 activation in response to interleukin-6 receptor and epidermal growth factor receptor signaling through JAK and/or MEK in head and neck squamous cell carcinomas.. Mol Cancer Ther.

[B34] Gerdes MJ, Myakishev M, Frost NA, Rishi V, Moitra J, Acharya A, Levy MR, Park SW, Glick A, Yuspa SH (2006). Activator protein-1 activity regulates epithelial tumor cell identity.. Cancer Res.

[B35] Cao XM, Koski RA, Gashler A, McKiernan M, Morris CF, Gaffney R, Hay RV, Sukhatme VP (1990). Identification and characterization of the Egr-1 gene product, a DNA-binding zinc finger protein induced by differentiation and growth signals.. Mol Cell Biol.

[B36] Worden B, Yang XP, Lee TL, Bagain L, Yeh NT, Cohen JG, Van Waes C, Chen Z (2005). Hepatocyte growth factor/scatter factor differentially regulates expression of proangiogenic factors through Egr-1 in head and neck squamous cell carcinoma.. Cancer Res.

[B37] Dong G, Loukinova E, Chen Z, Gangi L, Chanturita TI, Liu ET, Van Waes C (2001). Molecular profiling of transformed and metastatic murine squamous carcinoma cells by differential display and cDNA microarray reveals altered expression of multiple genes related to growth, apoptosis, angiogenesis, and the NF-kappaB signal pathway.. Cancer Res.

[B38] Loercher A, Lee TL, Ricker JL, Howard A, Geoghegen J, Chen Z, Sunwoo JB, Sitcheran R, Chuang EY, Mitchell JB (2004). Nuclear factor-kappaB is an important modulator of the altered gene expression profile and malignant phenotype in squamous cell carcinoma.. Cancer Res.

[B39] Zhang MQ (1999). Large-scale gene expression data analysis: a new challenge to computational biologists.. Genome Res.

[B40] Brazma A, Vilo J (2000). Gene expression data analysis.. FEBS Lett.

[B41] Bradford CR, Zhu S, Ogawa H, Ogawa T, Ubell M, Narayan A, Johnson G, Wolf GT, Fisher SG, Carey TE (2003). P53 mutation correlates with cisplatin sensitivity in head and neck squamous cell carcinoma lines.. Head Neck.

[B42] Chen Z, Colon I, Ortiz N, Callister M, Dong G, Pegram MY, Arosarena O, Strome S, Nicholson JC, Van Waes C (1998). Effects of interleukin-1alpha, interleukin-1 receptor antagonist, and neutralizing antibody on proinflammatory cytokine expression by human squamous cell carcinoma lines.. Cancer Res.

[B43] Kato T, Duffey DC, Ondrey FG, Dong G, Chen Z, Cook JA, Mitchell JB, Van Waes C (2000). Cisplatin and radiation sensitivity in human head and neck squamous carcinomas are independently modulated by glutathione and transcription factor NF-kappaB.. Head Neck.

[B44] Lee TL, Yang X, Yan B, Freidman J, Duggal P, Bagain L, Geoghegan J, Dong G, Yeh TN, Wang J (2007). A novel NF-kappaB gene signature is differentially expressed in head and neck squamous cell carcinomas in association with TP53 status.. Clin Cancer Res.

[B45] Simon R, Lam A, Li M, Ngan M, Menenzes M, Zhao Y (2007). Analysis of gene expression data using BRB-Array Tools.. Cancer Informatics.

[B46] Draghici S, Khatri P, Martins RP, Ostermeier GC, Krawetz SA (2003). Global functional profiling of gene expression.. Genomics.

[B47] Loukinova E, Chen Z, Van Waes C, Dong G (2001). Expression of proangiogenic chemokine Gro 1 in low and high metastatic variants of Pam murine squamous cell carcinoma is differentially regulated by IL-1alpha, EGF and TGF-beta1 through NF-kappaB dependent and independent mechanisms.. Int J Cancer.

[B48] Dong G, Chen Z, Li ZY, Yeh NT, Bancroft CC, Van Waes C (2001). Hepatocyte growth factor/scatter factor-induced activation of MEK and PI3K signal pathways contributes to expression of proangiogenic cytokines interleukin-8 and vascular endothelial growth factor in head and neck squamous cell carcinoma.. Cancer Res.

[B49] Bancroft CC, Chen Z, Dong G, Sunwoo JB, Yeh N, Park C, Van Waes C (2001). Coexpression of proangiogenic factors IL-8 and VEGF by human head and neck squamous cell carcinoma involves coactivation by MEK-MAPK and IKK-NF-kappaB signal pathways.. Clin Cancer Res.

[B50] Baron V, Adamson ED, Calogero A, Ragona G, Mercola D (2006). The transcription factor Egr1 is a direct regulator of multiple tumor suppressors including TGFbeta1, PTEN, p53, and fibronectin.. Cancer Gene Ther.

[B51] el-Deiry WS (1998). Regulation of p53 downstream genes.. Semin Cancer Biol.

[B52] Somasundaram K (2000). Tumor suppressor p53: regulation and function.. Front Biosci.

[B53] Liu HS, Pan CE, Liu QG, Yang W, Liu XM (2003). Effect of NF-kappaB and p38 MAPK in activated monocytes/macrophages on pro-inflammatory cytokines of rats with acute pancreatitis.. World J Gastroenterol.

[B54] Morgenstern B, Frech K, Dress A, Werner T (1998). DIALIGN: finding local similarities by multiple sequence alignment.. Bioinformatics.

[B55] Werner T, Fessele S, Maier H, Nelson PJ (2003). Computer modeling of promoter organization as a tool to study transcriptional coregulation.. Faseb J.

[B56] Hunter KD, Parkinson EK, Harrison PR (2005). Profiling early head and neck cancer.. Nat Rev Cancer.

[B57] Webster GA, Perkins ND (1999). Transcriptional cross talk between NF-kappaB and p53.. Mol Cell Biol.

[B58] Rocha S, Martin AM, Meek DW, Perkins ND (2003). p53 represses cyclin D1 transcription through down regulation of Bcl-3 and inducing increased association of the p52 NF-kappaB subunit with histone deacetylase 1.. Mol Cell Biol.

[B59] Rocha S, Garrett MD, Campbell KJ, Schumm K, Perkins ND (2005). Regulation of NF-kappaB and p53 through activation of ATR and Chk1 by the ARF tumour suppressor.. Embo J.

[B60] Raman V, Martensen SA, Reisman D, Evron E, Odenwald WF, Jaffee E, Marks J, Sukumar S (2000). Compromised HOXA5 function can limit p53 expression in human breast tumours.. Nature.

[B61] Kang JH, Kim SJ, Noh DY, Park IA, Choe KJ, Yoo OJ, Kang HS (2001). Methylation in the p53 promoter is a supplementary route to breast carcinogenesis: correlation between CpG methylation in the p53 promoter and the mutation of the p53 gene in the progression from ductal carcinoma in situ to invasive ductal carcinoma.. Lab Invest.

[B62] Rocco JW, Leong CO, Kuperwasser N, DeYoung MP, Ellisen LW (2006). p63 mediates survival in squamous cell carcinoma by suppression of p73-dependent apoptosis.. Cancer Cell.

[B63] Luger K, Rechsteiner TJ, Flaus AJ, Waye MM, Richmond TJ (1997). Characterization of nucleosome core particles containing histone proteins made in bacteria.. J Mol Biol.

[B64] Bassing CH, Chua KF, Sekiguchi J, Suh H, Whitlow SR, Fleming JC, Monroe BC, Ciccone DN, Yan C, Vlasakova K (2002). Increased ionizing radiation sensitivity and genomic instability in the absence of histone H2AX.. Proc Natl Acad Sci USA.

[B65] Martini EM, Keeney S, Osley MA (2002). A role for histone H2B during repair of UV-induced DNA damage in *Saccharomyces cerevisiae*.. Genetics.

[B66] Zhang Y (2003). Transcriptional regulation by histone ubiquitination and deubiquitination.. Genes Dev.

[B67] Minsky N, Oren M (2004). The RING domain of Mdm2 mediates histone ubiquitylation and transcriptional repression.. Mol Cell.

[B68] Yu X, Caltagarone J, Smith MA, Bowser R (2005). DNA damage induces cdk2 protein levels and histone H2B phosphorylation in SH-SY5Y neuroblastoma cells.. J Alzheimers Dis.

[B69] Espinosa JM, Emerson BM (2001). Transcriptional regulation by p53 through intrinsic DNA/chromatin binding and site-directed cofactor recruitment.. Mol Cell.

[B70] Chen Z, Malhotra PS, Thomas GR, Ondrey FG, Duffey DC, Smith CW, Enamorado I, Yeh NT, Kroog GS, Rudy S (1999). Expression of proinflammatory and proangiogenic cytokines in patients with head and neck cancer.. Clin Cancer Res.

[B71] Chung CH, Parker JS, Ely K, Carter J, Yi Y, Murphy BA, Ang KK, El-Naggar AK, Zanation AM, Cmelak AJ (2006). Gene expression profiles identify epithelial-to-mesenchymal transition and activation of nuclear factor-{kappa}b signaling as characteristics of a high-risk head and neck squamous cell carcinoma.. Cancer Res.

[B72] Welty SE, Rivera JL, Elliston JF, Smith CV, Zeb T, Ballantyne CM, Montgomery CA, Hansen TN (1993). Increases in lung tissue expression of intercellular adhesion molecule-1 are associated with hyperoxic lung injury and inflammation in mice.. Am J Respir Cell Mol Biol.

[B73] Colletti LM, Cortis A, Lukacs N, Kunkel SL, Green M, Strieter RM (1998). Tumor necrosis factor up-regulates intercellular adhesion molecule 1, which is important in the neutrophil-dependent lung and liver injury associated with hepatic ischemia and reperfusion in the rat.. Shock.

[B74] Kacimi R, Karliner JS, Koudssi F, Long CS (1998). Expression and regulation of adhesion molecules in cardiac cells by cytokines: response to acute hypoxia.. Circ Res.

[B75] Stanciu LA, Djukanovic R (1998). The role of ICAM-1 on T-cells in the pathogenesis of asthma.. Eur Respir J.

[B76] Bavendiek U, Libby P, Kilbride M, Reynolds R, Mackman N, Schonbeck U (2002). Induction of tissue factor expression in human endothelial cells by CD40 ligand is mediated via activator protein 1, nuclear factor kappa B, and Egr-1.. J Biol Chem.

[B77] Thyss R, Virolle V, Imbert V, Peyron JF, Aberdam D, Virolle T (2005). NF-kappaB/Egr-1/Gadd45 are sequentially activated upon UVB irradiation to mediate epidermal cell death.. Embo J.

[B78] Squarize CH, Castilho RM, Sriuranpong V, Pinto DS, Gutkind JS (2006). Molecular cross-talk between the NFkappaB and STAT3 signaling pathways in head and neck squamous cell carcinoma.. Neoplasia.

[B79] Yang J, Liao X, Agarwal MK, Barnes L, Auron PE, Stark GR (2007). Unphosphorylated STAT3 accumulates in response to IL-6 and activates transcription by binding to NF{kappa}B.. Genes Dev.

[B80] Dong G, Lee TL, Yeh NT, Geoghegan J, Van Waes C, Chen Z (2004). Metastatic squamous cell carcinoma cells that overexpress c-Met exhibit enhanced angiogenesis factor expression, scattering and metastasis in response to hepatocyte growth factor.. Oncogene.

[B81] Gumucio DL, Heilstedt-Williamson H, Gray TA, Tarle SA, Shelton DA, Tagle DA, Slightom JL, Goodman M, Collins FS (1992). Phylogenetic footprinting reveals a nuclear protein which binds to silencer sequences in the human gamma and epsilon globin genes.. Mol Cell Biol.

[B82] Zhang Z, Gerstein M (2003). Of mice and men: phylogenetic footprinting aids the discovery of regulatory elements.. J Biol.

[B83] Zhang YF, Homer C, Edwards SJ, Hananeia L, Lasham A, Royds J, Sheard P, Braithwaite AW (2003). Nuclear localization of Y-box factor YB1 requires wild-type p53.. Oncogene.

[B84] Sinha S, Kim IS, Sohn KY, de Crombrugghe B, Maity SN (1996). Three classes of mutations in the A subunit of the CCAAT-binding factor CBF delineate functional domains involved in the three-step assembly of the CBF-DNA complex.. Mol Cell Biol.

[B85] Maity SN, de Crombrugghe B (1998). Role of the CCAAT-binding protein CBF/NF-Y in transcription.. Trends Biochem Sci.

[B86] Mantovani R (1999). The molecular biology of the CCAAT-binding factor NF-Y.. Gene.

[B87] Kim IS, Sinha S, de Crombrugghe B, Maity SN (1996). Determination of functional domains in the C subunit of the CCAAT-binding factor (CBF) necessary for formation of a CBF-DNA complex: CBF-B interacts simultaneously with both the CBF-A and CBF-C subunits to form a heterotrimeric CBF molecule.. Mol Cell Biol.

[B88] Sinha S, Maity SN, Lu J, de Crombrugghe B (1995). Recombinant rat CBF-C, the third subunit of CBF/NFY, allows formation of a protein-DNA complex with CBF-A and CBF-B and with yeast HAP2 and HAP3.. Proc Natl Acad Sci USA.

[B89] Romier C, Cocchiarella F, Mantovani R, Moras D (2003). The NF-YB/NF-YC structure gives insight into DNA binding and transcription regulation by CCAAT factor NF-Y.. J Biol Chem.

[B90] Imbriano C, Gurtner A, Cocchiarella F, Di Agostino S, Basile V, Gostissa M, Dobbelstein M, Del Sal G, Piaggio G, Mantovani R (2005). Direct p53 transcriptional repression: in vivo analysis of CCAAT-containing G2/M promoters.. Mol Cell Biol.

[B91] Oikawa T (2004). ETS transcription factors: possible targets for cancer therapy.. Cancer Sci.

[B92] Bassuk AG, Anandappa RT, Leiden JM (1997). Physical interactions between Ets and NF-kappaB/NFAT proteins play an important role in their cooperative activation of the human immunodeficiency virus enhancer in T cells.. J Virol.

[B93] Graves BJ, Petersen JM (1998). Specificity within the ets family of transcription factors.. Adv Cancer Res.

[B94] Oikawa T, Yamada T (2003). Molecular biology of the Ets family of transcription factors.. Gene.

[B95] Bai L, Logsdon C, Merchant JL (2002). Regulation of epithelial cell growth by ZBP-89: potential relevance in pancreatic cancer.. Int J Gastrointest Cancer.

[B96] Bai L, Merchant JL (2001). ZBP-89 promotes growth arrest through stabilization of p53.. Mol Cell Biol.

[B97] Remington MC, Tarle SA, Simon B, Merchant JL (1997). ZBP-89, a Kruppel-type zinc finger protein, inhibits cell proliferation.. Biochem Biophys Res Commun.

[B98] Borghaei RC, Rawlings PL, Javadi M, Woloshin J (2004). NF-kappaB binds to a polymorphic repressor element in the MMP-3 promoter.. Biochem Biophys Res Commun.

[B99] Keates AC, Keates S, Kwon JH, Arseneau KO, Law DJ, Bai L, Merchant JL, Wang TC, Kelly CP (2001). ZBP-89, Sp1, and nuclear factor-kappa B regulate epithelial neutrophil-activating peptide-78 gene expression in Caco-2 human colonic epithelial cells.. J Biol Chem.

[B100] Chen Z, Lee TL, Yang XP, Dong G, Loercher A, Van Waes C, Fisher PD (2007). cDNA microarray and bioinformatic analysis of nuclear factor-kappaB related genes in squamous cell carcinoma.. Cancer Genomics and Proteomics: Methods and Protocols.

[B101] Eisen MB, Spellman PT, Brown PO, Botstein D (1998). Cluster analysis and display of genome-wide expression patterns.. Proc Natl Acad Sci USA.

[B102] Stanford software: Significant Analysis of Microarray. http://www-stat.stanford.edu/~tibs/SAM/.

[B103] Saldanha AJ (2004). Java Treeview: extensible visualization of microarray data.. Bioinformatics.

[B104] Tempelman RJ (2005). Assessing statistical precision, power, and robustness of alternative experimental designs for two color microarray platforms based on mixed effects models.. Vet Immunol Immunopathol.

[B105] Wolfinger RD, Gibson G, Wolfinger ED, Bennett L, Hamadeh H, Bushel P, Afshari C, Paules RS (2001). Assessing gene significance from cDNA microarray expression data via mixed models.. J Comput Biol.

[B106] Gene Ontology: database. http://www.godatabase.org.

[B107] National Center for Biotechnology Information. http://www.ncbi.nlm.nih.gov.

[B108] Genomatix Software GmbH. http://www.genomatix.de.

[B109] Hoh J, Jin S, Parrado T, Edington J, Levine AJ, Ott J (2002). The p53MH algorithm and its application in detecting p53-responsive genes.. Proc Natl Acad Sci USA.

[B110] Bajic VB, Tan SL, Chong A, Tang S, Strom A, Gustafsson JA, Lin CY, Liu ET (2003). Dragon ERE Finder version 2: A tool for accurate detection and analysis of estrogen response elements in vertebrate genomes.. Nucleic Acids Res.

[B111] Benos PV, Lapedes AS, Stormo GD (2002). Probabilistic code for DNA recognition by proteins of the EGR family.. J Mol Biol.

[B112] Cartharius K, Frech K, Grote K, Klocke B, Haltmeier M, Klingenhoff A, Frisch M, Bayerlein M, Werner T (2005). MatInspector and beyond: promoter analysis based on transcription factor binding sites.. Bioinformatics.

[B113] Quandt K, Frech K, Karas H, Wingender E, Werner T (1995). MatInd and MatInspector: new fast and versatile tools for detection of consensus matches in nucleotide sequence data.. Nucleic Acids Res.

[B114] Hughes JD, Estep PW, Tavazoie S, Church GM (2000). Computational identification of cis-regulatory elements associated with groups of functionally related genes in *Saccharomyces cerevisiae*.. J Mol Biol.

[B115] Werner T (2003). Promoters can contribute to the elucidation of protein function.. Trends Biotechnol.

[B116] Dohr S, Klingenhoff A, Maier H, Hrabe de Angelis M, Werner T, Schneider R (2005). Linking disease-associated genes to regulatory networks via promoter organization.. Nucleic Acids Res.

