# Supplementary material for: Genome-wide identification of novel expression signatures reveal distinct patterns and prevalence of binding motifs for p53, nuclear factor-κB and other signal transcription factors in head and neck squamous cell carcinoma
Source: Genome Biol. 2007 May 11;8(5):R78. doi: 10.1186/gb-2007-8-5-r78 (PMC1929156; doi:10.1186/gb-2007-8-5-r78)
Supplement: Additional data file 1 — Shown is a list of genes in cluster C (over-expressed in UM-SCC cells). [file gb-2007-8-5-r78-S1.pdf]

Supplemental Table S1. Gene list of cluster C overexpressed in HNSCC

Symbol [1]	Gene description	Cytogenetic Location	Fold Change		
			Tumor[1]	wt p53-like[2]	mt p53[3]
CRABP2	cellular retinoic acid binding protein 2	1q21.3	2.89	1.61	4.16
IL22RA1	interleukin 22 receptor	1p36.11	3.13	2.30	3.96
CDW52	CDW52 antigen (CAMPATH-1 antigen)	1p36	2.22	1.88	2.56
RARRES3	retinoic acid receptor responder (tazarotene induced) 3	11q23	3.31	2.43	4.18
APOL1	apolipoprotein L, 1	22q13.1	3.27	3.04	3.49
UBE2D4	ubiquitin-conjugating enzyme HBUCE1	7p13	2.78	2.66	2.90
APOL3	apolipoprotein L, 3	22q13.1	3.63	3.71	3.55
CGI-49	CGI-49 protein	1q44	2.07	2.23	1.91
SGCE	sarcoglycan, epsilon	7q21-q22	2.03	1.96	2.09
ALDH1A1	aldehyde dehydrogenase 1 family, member A1	9q21	6.32	2.21	10.43
NQO1	NAD(P)H dehydrogenase, quinone 1	16q22.1	2.51	2.26	2.76
ALP	alpha-actinin-2-associated LIM protein	4q35	2.36	2.12	2.60
FOXE1	forkhead box E1 (thyroid transcription factor 2)	9q22	2.06	1.91	2.21
CRIP1	cysteine-rich protein 1 (intestinal)	7q11.23	2.22	1.72	2.72
KCNJ8	potassium inwardly-rectifying channel, subfamily J, member 8	12p11.23	2.03	2.16	1.90
ISYNA1	myo-inositol 1-phosphate synthase A1	19p13.12	2.20	2.17	2.23
PIK3R3	phosphoinositide-3-kinase, regulatory subunit, polypeptide 3 (p55, gamma)	1pter-p32.1	2.32	1.86	2.77
EPS8	epidermal growth factor receptor pathway substrate 8	12q23-q24	2.52	3.29	1.75
SIM2	single-minded homolog 2 (Drosophila)	21q22.13	2.81	2.79	2.83
MAPK8IP1	mitogen-activated protein kinase 8 interacting protein 1	11p12-p11.2	2.15	2.15	2.16
CBX6	chromobox homolog 6	22q13.1	2.17	1.87	2.46
LAMP3	lysosomal-associated membrane protein 3	3q26.3-q27	3.09	2.27	3.91
MYO5B	myosin VB	18q21	2.49	2.01	2.97
TSPAN13	Tetraspanin 13	7p21.1	2.24	2.20	2.28
IMP-3	IGF-II mRNA-binding protein 3	7p11	1.97	1.91	2.03
HOXA13	homeo box A13	7p15-p14	2.01	1.69	2.33
TARBP1	TAR (HIV) RNA binding protein 1	1p	1.76	1.51	2.00
GBAS	glioblastoma amplified sequence	7p12	1.87	1.69	2.04
CEP1	centrosomal protein 1	9q33-q34	2.47	2.05	2.88
TAX1BP1	Tax1 (human T-cell leukemia virus type I) binding protein 1	7p15	2.00	2.08	1.91
HIBADH	3-hydroxyisobutyrate dehydrogenase	7p15.2	2.04	2.29	1.79
CLDN4	claudin 4	7q11.23	3.19	3.41	2.97
CLDN3	claudin 3	7q11.23	6.61	7.74	5.49
MDK	midkine (neurite growth-promoting factor 2)	11p11.2	2.53	2.35	2.72
KIAA1102	KIAA1102 protein	4p13	5.14	4.47	5.81
MEIS2	Meis1, myeloid ecotropic viral integration site 1 homolog 2 (mouse)	15q13.2	2.24	1.81	2.66
NFIL3	nuclear factor, interleukin 3 regulated	9q22	1.94	1.85	2.03
LMO7	LIM domain only 7	13q21.33	3.47	2.69	4.26
HRASLS3	HRAS-like suppressor 3	11q12.3	2.11	1.96	2.27
RPS6KA5	ribosomal protein S6 kinase, 90kD, polypeptide 5	14q31-q32.1	2.35	1.75	2.94
RAB22A	RAB22A, member RAS oncogene family	20q13.32	2.37	1.72	3.02
DNM1	dynamin 1	9q34	1.86	1.42	2.31
GPX4	glutathione peroxidase 4 (phospholipid hydroperoxidase)	19p13.3	1.76	1.31	2.20
BCAN	chondroitin sulfate proteoglycan BEHAB/brevican	1q31	5.83	2.78	8.87
NCOA3	nuclear receptor coactivator 3	20q12	1.78	1.56	2.00
CCND1	cyclin D1 (PRAD1: parathyroid adenomatosis 1)	11q13	2.38	1.73	3.04
GDA	guanine deaminase	9q21.11-21.33	4.80	3.18	6.43
CHK	choline kinase	11cen-q12.1	2.70	2.53	2.86
CCS	copper chaperone for superoxide dismutase	11q13	2.05	1.84	2.25
FBXL11	F-box and leucine-rich repeat protein 11	11q13.2	2.68	2.56	2.81
CIT	citron (rho-interacting, serine/threonine kinase 21)	12q	2.42	2.28	2.57

NDUFV1	NADH dehydrogenase (ubiquinone) flavoprotein 1 (51kD)	11q13	1.98	1.93	2.03
GPR56	G protein-coupled receptor 56	16q13	1.76	1.51	2.00
Cluster C1					
**NOT56L**	**Not56 (D. melanogaster)-like protein**	**3q26.1-q26.33**	**1.83**	**1.65**	**2.01**
**OGFR**	**opioid growth factor receptor**	**20q13.3**	**1.85**	**1.55**	**2.16**
**HIST1H2AL**	**H2A histone family, member I**	**6p22-p21.3**	**2.08**	**1.57**	**2.60**
**IFI30**	**interferon, gamma-inducible protein 30**	**19p13.1**	**2.02**	**1.74**	**2.30**
**ERBB3**	**v-erb-b2 erythroblastic leukemia viral oncogene homolog 3**	**12q13**	**1.74**	**1.46**	**2.02**
**ELTD1**	**EGF-TM7-latrophilin-related protein**	**1p33-p32**	**1.77**	**1.41**	**2.13**
**LBP-32**	**LBP protein 32**	**2p25.2**	**2.27**	**1.96**	**2.58**
**IGFBP3**	**insulin-like growth factor binding protein 3**	**7p13-p12**	**33.12**	**15.91**	**50.34**
**HINT1**	**histidine triad nucleotide-binding protein**	**5q31.2**	**1.97**	**1.78**	**2.17**
**PIN1**	**protein (peptidyl-prolyl cis/trans isomerase) NIMA-interacting 1**	**19p13**	**1.84**	**1.42**	**2.25**
**NUP214**	**nucleoporin 214kD (CAIN)**	**9q34.1**	**1.94**	**1.63**	**2.25**
**REA**	**B-cell associated protein**	**12p13**	**1.94**	**1.59**	**2.28**
**SULT1A1**	**phenol-preferring phenol sulfotransferase1**	**16p12.1**	**2.07**	**1.74**	**2.41**
**GCA**	**grancalcin, EF-hand calcium binding protein**	**2q24.3**	**2.11**	**1.60**	**2.61**
**CREG**	**cellular repressor of E1A-stimulated genes**	**1q24**	**2.60**	**2.03**	**3.16**
**P5CR2**	**pyrroline 5-carboxylate reductase isoform**	**1q42.13**	**1.65**	**1.26**	**2.03**
**SCHIP1**	**schwannomin interacting protein 1**	**3q26.1**	**2.14**	**1.60**	**2.69**
**C20orf99**	**chromosome 20 open reading frame 99**	**20p13-p12.2**	**1.92**	**1.45**	**2.38**
**RISC**	**serine carboxypeptidase 1**	**17q23.2**	**2.01**	**1.72**	**2.29**
**ALDH3A2**	**aldehyde dehydrogenase 3 family, member A2**	**17p11.2**	**1.74**	**1.36**	**2.11**
**RALGDS**	**ral guanine nucleotide dissociation stimulator**	**9q34**	**1.99**	**1.41**	**2.58**
**TSC1**	**tuberous sclerosis 1**	**9q34**	**1.72**	**1.44**	**2.00**
**PDK2**	**pyruvate dehydrogenase kinase, isoenzyme 2**	**17q21.33**	**2.31**	**1.78**	**2.84**
**HDAC5**	**histone deacetylase 5**	**17q21**	**2.02**	**1.28**	**2.76**
**RABGAP1**	**RAB GTPase activating protein 1**	**9q33.2-q33.3**	**1.76**	**1.34**	**2.19**
**CALR**	**calreticulin**	**19p13.3-p13.2**	**2.21**	**1.39**	**3.03**
**SNCAIP**	**synuclein, alpha interacting protein (synphilin)**	**5q23.1-q23.3**	**1.80**	**1.23**	**2.37**
**ZNF297B**	**zinc finger protein 297B**	**9p24.1-q22.33**	**1.61**	**1.18**	**2.05**
**NFATC3**	**cytoplasmic nuclear factor of activated T-cells 3**	**16q13-q24**	**1.64**	**1.28**	**2.01**
**MAGEF1**	**MAGEF1 protein**	**3q13**	**2.94**	**1.80**	**4.08**
**F8**	**coagulation factor VIII**	**Xq28**	**2.06**	**1.43**	**2.70**
**SIRT7**	**silencing information regulator 2-like**	**17q25**	**1.68**	**1.26**	**2.09**
**SURF1**	**surfeit 1**	**9q33-q34**	**1.99**	**1.83**	**2.15**
**HEXA**	**hexosaminidase A (alpha polypeptide)**	**15q23-q24**	**1.88**	**1.70**	**2.07**
**GOLGA1**	**golgi autoantigen, golgin subfamily a, 1**	**9q34.11**	**2.02**	**1.78**	**2.27**
**GAA**	**acid alpha-glucosidase**	**17q25.2-q25.3**	**3.50**	**2.64**	**4.35**
**DAZAP2**	**DAZ associated protein 2**	**2q33-q34**	**2.01**	**1.63**	**2.39**
**MCCC1**	**methylcrotonoyl-Coenzyme A carboxylase 1 (alpha)**	**3q27**	**3.02**	**2.17**	**3.88**
**DVL3**	**dishevelled, dsh homolog 3 (Drosophila)**	**3q27**	**2.18**	**1.62**	**2.75**
**ALDH3B2**	**aldehyde dehydrogenase 3 family, member B2**	**11q13**	**2.00**	**1.89**	**2.10**
**NCOA1**	**nuclear receptor coactivator 1**	**2p23**	**1.79**	**1.53**	**2.04**
**HOXD9**	**homeo box D9**	**2q31-q37**	**2.41**	**1.83**	**2.99**
**HOXD10**	**homeo box D10**	**2q31.1**	**2.31**	**2.01**	**2.62**
**PLA2G4B**	**phospholipase A2, group IVB (cytosolic)**	**15q11.2-q21.3**	**1.70**	**1.05**	**2.34**
**EPHX1**	**epoxide hydrolase 1, microsomal (xenobiotic)**	**1q42.1**	**2.65**	**1.46**	**3.84**
**MPHOSPH9**	**M-phase phosphoprotein 9**	**12q24.31**	**1.63**	**1.23**	**2.02**
BDG29	BDG-29 proten	16q24.2	1.74	1.37	2.11
TFRC	transferrin receptor (p90, CD71)	3q26.2-qter	2.97	3.38	2.56
HSUP1	Similar to RPE-spondin	20q13.13	2.52	2.74	2.30
PTGES	prostaglandin E synthase	9q34.3	2.70	2.93	2.47
ADPRTL3	ADP-ribosyltransferase (NAD+; poly (ADP-ribose) polymerase)-like 3	3p22.2-p21.1	4.52	5.56	3.48

PODXL	podocalyxin-like	7q32-q33	4.16	3.56	4.76
GPR109B	G protein-coupled receptor 109B	12q24.31	3.12	2.94	3.30
USP11	ubiquitin specific protease 11	1q21	2.51	2.18	2.85
CDT1	DNA replication factor	16q24	2.07	1.76	2.38
EIF4G1	eukaryotic translation initiation factor 4 gamma, 1	3q27-qter	3.26	2.93	3.60
ANXA13	annexin A13	8q24.1-q24.2	2.24	2.04	2.44
ADA	adenosine deaminase	20q12-q13.11	2.26	2.16	2.36
PCCB	propionyl Coenzyme A carboxylase, beta polypeptide	3q21-q22	2.95	2.73	3.16
C20orf104	chromosome 20 open reading frame 104	20q11.22-q11.23	2.76	2.56	2.96
PTGS1	prostaglandin-endoperoxide synthase 1 (prostaglandin G/H synthase and cyclooxygenase)	9q32-q33.3	2.71	2.46	2.97
DHODH	dihydroorotate dehydrogenase	16q22	2.53	2.36	2.69
DOLPP1	Dolichyl pyrophosphate phosphatase 1	9q34.1	2.19	1.83	2.55
NEU1	sialidase 1 (lysosomal sialidase)	6p21.3	1.90	1.67	2.12
USP13	ubiquitin specific protease 13 (isopeptidase T-3)	3q26.2-q26.3	2.01	2.38	1.65
MSX1	msh homeo box homolog 1 (Drosophila)	4p16.3-p16.1	2.46	2.97	1.95
CYB561	cytochrome b-561	17q11-qter	2.18	2.59	1.77
TBX5	T-box 5	12q24.1	2.00	2.33	1.67
APBA2BP	amyloid beta (A4) precursor protein-binding, family A, member 2 binding protein	20q11.22	1.96	2.01	1.91
HIST1H4C	H4 histone family, member G	6p21.3	2.73	3.25	2.21
HIST1H4B	H4 histone family, member I	6p21.3	1.96	2.21	1.70
HIST1H4E	H4 histone family, member J	6p21.3	1.90	2.01	1.79
SMC2L1	SMC2 structural maintenance of chromosomes 2-like 1 (yeast)	9q31.1	1.87	2.12	1.62
B4GALT5	UDP-Gal:betaGlcNAc beta 1,4- galactosyltransferase, polypeptide 5	20q13.1-q13.2	2.03	1.95	2.11
ESPL1	extra spindle poles like 1 (S. cerevisiae)	12q	1.87	1.72	2.02
Cluster C2					
**TRAP95**	**thyroid hormone receptor-associated protein, 95-kD subunit**	**19p13.3**	**2.93**	**3.71**	**2.16**
**BAG2**	**BCL2-associated athanogene 2**	**6p12.3-p11.2**	**4.43**	**5.79**	**3.06**
**RAMP2**	**receptor (calcitonin) activity modifying protein 2**	**17q12-q21.1**	**1.65**	**2.07**	**1.24**
**HOXB9**	**homeo box B9**	**17q21.3**	**2.18**	**2.67**	**1.70**
**TK1**	**thymidine kinase 1, soluble**	**17q23.2-q25.3**	**2.48**	**2.58**	**2.37**
**RECQL4**	**RecQ protein-like 4**	**8q24.3**	**2.21**	**2.43**	**1.98**
**CRIP2**	**cysteine-rich protein 2**	**14q32.3**	**2.54**	**2.81**	**2.27**
**EPHB2**	**EphB2**	**1p36.1-p35**	**2.02**	**2.44**	**1.61**
**OAZIN**	**Antizyme inhibitor 1**	**8q22.3**	**1.89**	**2.21**	**1.57**
**ATAD2**	**ATPase family, AAA domain containing 2**	**8q24.13**	**2.56**	**3.06**	**2.07**
**FBXO5**	**F-box only protein 5**	**6q25-q26**	**1.96**	**2.37**	**1.54**
**CDC45L**	**CDC45 cell division cycle 45-like (S. cerevisiae)**	**22q11.21**	**2.21**	**2.88**	**1.54**
**MYBL2**	**v-myb myeloblastosis viral oncogene homolog (avian)-like 2**	**20q13.1**	**2.71**	**3.39**	**2.03**
**ORC1L**	**origin recognition complex, subunit 1-like (yeast)**	**1p32**	**1.71**	**2.13**	**1.30**
**NASP**	**nuclear autoantigenic sperm protein (histone-binding)**	**1p34.1**	**1.87**	**2.40**	**1.34**
**KIF4A**	**kinesin family member 4A**	**Xq13.1**	**1.80**	**2.16**	**1.44**
**KIF2C**	**kinesin-like 6 (mitotic centromere-associated kinesin)**	**1p34.1**	**3.14**	**4.18**	**2.10**
**FBXL14**	**F-box and leucine-rich repeat protein 14**	**12p13.33**	**2.10**	**2.52**	**1.69**
**RNPS1**	**RNA binding protein S1, serine-rich domain**	**16p13.3**	**2.54**	**2.78**	**2.31**
**RNPC1**	**RNA-binding region (RNP1, RRM) containing 1**	**20q13.31**	**2.52**	**2.86**	**2.18**
**DDX11**	**DEAD/H (Asp-Glu-Ala-Asp/His) box polypeptide 11**	**12p11**	**2.45**	**2.86**	**2.04**
**CNAP1**	**chromosome condensation-related SMC-associated protein 1**	**12p13.3**	**2.64**	**2.88**	**2.40**
**UBE2C**	**ubiquitin-conjugating enzyme E2C**	**20q13.12**	**2.80**	**3.18**	**2.43**
**C20orf1**	**chromosome 20 open reading frame 1**	**20q11.2**	**2.42**	**2.84**	**2.00**
**RAI3**	**retinoic acid induced 3**	**12p13-p12.3**	**3.36**	**3.93**	**2.79**
**TBCD**	**tubulin-specific chaperone d**	**17q25.3**	**1.82**	**2.09**	**1.55**
**RPA3**	**replication protein A3 (14kD)**	**7p22**	**2.43**	**2.96**	**1.90**
**PTPRA**	**protein tyrosine phosphatase, receptor type, A**	**20p13**	**2.69**	**3.36**	**2.02**

**CCNB2**	**cyclin B2**	**15q21.2**	**2.62**	**2.81**	**2.43**
CPSF5	cleavage and polyadenylation specific factor 5, 25 kD subunit	16q12.2	1.71	2.01	1.42
ARAP3	ARF-GAP, RHO-GAP, ankyrin repeat and plekstrin homology domains-containing protein 3	5q31.3	2.00	2.44	1.56
C20orf24	chromosome 20 open reading frame 24	20q11.23	2.50	2.91	2.09
GLE1L	GLE1 RNA export mediator-like (yeast)	9q34.13	2.05	2.08	2.02
POLE3	polymerase (DNA directed), epsilon 3 (p17 subunit)	9q33	2.01	2.09	1.92
MCM2	MCM2 minichromosome maintenance deficient 2, mitotin (S. cerevisiae)	3q21	2.17	2.40	1.93
HSPC176	Hematopoietic stem/progenitor cells 176	16q24.3	1.92	2.03	1.80
TROAP	trophinin associated protein (tastin)	12q13.12	1.83	2.13	1.53
Cluster C3					
**BAT8**	**HLA-B associated transcript 8**	**6p21.3**	**1.99**	**2.05**	**1.93**
**DEK**	**DEK oncogene (DNA binding)**	**6p23**	**2.17**	**1.99**	**2.36**
**ZNF266**	**zinc finger protein 266**	**19p13.2**	**2.00**	**1.91**	**2.10**
**TOPBP1**	**topoisomerase (DNA) II binding protein**	**3q22.2**	**2.23**	**1.97**	**2.49**
**ITPKA**	**inositol 1,4,5-trisphosphate 3-kinase A**	**15q14-q21**	**2.48**	**2.13**	**2.83**
**UBXD5**	**Socius**	**1p36.11**	**2.03**	**2.11**	**1.96**
**ALDH5A1**	**aldehyde dehydrogenase 5 family, member A1**	**6p22**	**2.03**	**2.18**	**1.88**
**AKNA**	**AT-hook transcription factor AKNA**	**9q32**	**2.29**	**2.50**	**2.08**
**ARL6IP**	**ADP-ribosylation factor-like 6**	**16p12-p11.2**	**2.12**	**2.31**	**1.93**
**PRIM1**	**primase, polypeptide 1 (49kD)**	**12q13**	**2.34**	**2.85**	**1.83**
**IFIT1**	**interferon-induced protein 56**	**10q25-q26**	**2.10**	**2.28**	**1.91**
**PPAP2C**	**phosphatidic acid phosphatase type 2C**	**19p13**	**4.29**	**4.01**	**4.56**
**FARP1**	**FERM, RhoGEF, and pleckstrin domain protein 1**	**13q32.2**	**2.55**	**2.57**	**2.52**
**MAL2**	**mal, T-cell differentiation protein 2**	**8q23**	**3.68**	**3.67**	**3.69**
**ETV1**	**ets variant gene 1**	**7p21.3**	**4.10**	**4.33**	**3.88**
**VPS45A**	**vacuolar protein sorting 45A (yeast)**	**1q21-q22**	**2.09**	**1.73**	**2.44**
**TMOD1**	**tropomodulin**	**9q22.3**	**1.90**	**1.58**	**2.21**
**RAE1**	**RAE1 RNA export 1 homolog (S. pombe)**	**20q13.31**	**2.35**	**2.37**	**2.32**
**VPS41**	**vacuolar protein sorting 41 (yeast)**	**7p14-p13**	**1.99**	**1.91**	**2.07**
**IVD**	**isovaleryl Coenzyme A dehydrogenase**	**15q14-q15**	**2.47**	**2.38**	**2.56**
**CIA30**	**CGI-65 protein**	**15q11.2-q21.3**	**2.78**	**2.93**	**2.63**
**SEMA4D**	**semaphorin 4D**	**9q22-q31**	**9.55**	**9.23**	**9.87**
**TNXB**	**tenascin XB**	**6p21.3**	**15.95**	**13.79**	**18.11**
**C20orf11**	**chromosome 20 open reading frame 11**	**20q13.33**	**2.15**	**2.11**	**2.20**
**KEAP1**	**Kelch-like ECH-associated protein 1**	**19p13.2**	**2.26**	**2.17**	**2.35**
**ANKT**	**nucleolar protein ANKT**	**15q13.3**	**2.53**	**2.26**	**2.80**
**TOP2A**	**topoisomerase (DNA) II alpha (170kD)**	**17q21-q22**	**2.13**	**2.10**	**2.16**
**RFC4**	**replication factor C 4**	**3q27**	**2.00**	**2.00**	**2.01**
**DHX35**	**DEAD/H box polypeptide 35**	**20pter-q12**	**1.92**	**2.05**	**1.80**
**PIGT**	**Phosphatidylinositol glycan, class T**	**20q12-q13.12**	**2.10**	**1.95**	**2.25**
**ERGIC3**	**ERGIC and golgi 3**	**20pter-q12**	**1.99**	**2.10**	**1.87**
**TAF7**	**TAF7 RNA polymerase II, 55 kD**	**5q31**	**2.11**	**2.06**	**2.16**
**F8A1**	**coagulation factor VIII-associated protein**	**Xq28**	**2.16**	**2.07**	**2.24**
**SENP2**	**sentrin-specific protease**	**3q27.2**	**2.06**	**1.82**	**2.30**
**GOLGA1**	**golgi autoantigen, golgin subfamily a, 1**	**9q34.11**	**2.02**	**1.78**	**2.27**
**TIMP2**	**tissue inhibitor of metalloproteinase 2**	**17q25**	**4.73**	**5.39**	**4.07**
**IL10RB**	**interleukin 10 receptor, beta**	**21q22.11**	**2.08**	**2.10**	**2.07**
**NEK2**	**NIMA-related kinase 2**	**1q32.2-q41**	**2.20**	**2.03**	**2.37**
**TNFAIP2**	**TNF, alpha-induced protein 2**	**14q32**	**9.21**	**11.33**	**7.09**
**PPGB**	**protective protein for beta-galactosidase**	**20q13.1**	**2.02**	**2.21**	**1.83**
**OVCA2**	**ovarian cancer gene-2 protein**	**17p13.3**	**1.97**	**1.84**	**2.10**
**HOXB7**	**homeo box B7**	**17q21-q22**	**2.17**	**2.08**	**2.27**
**SDR1**	**short-chain dehydrogenase/reductase 1**	**1p36.1**	**4.71**	**5.27**	**4.14**
**APIP**	**APAF1 interacting protein**	**11p13**	**2.01**	**2.17**	**1.84**

**HTATIP2**	**HIV-1 Tat interactive protein 2, 30 kD**	**11p15.1**	**2.54**	**2.46**	**2.62**
**PORIMIN**	**pro-oncosis receptor inducing membrane injury gene**	**11q22.1**	**1.97**	**2.10**	**1.84**
**C20orf35**	**chromosome 20 open reading frame 169**	**20q13.12**	**1.85**	**2.04**	**1.65**
**CLDN7**	**claudin 7**	**17p13**	**8.62**	**10.67**	**6.56**
**CGN**	**cingulin**	**1q21**	**2.42**	**2.90**	**1.93**
**TACSTD1**	**tumor-associated calcium signal transducer 1**	**4q**	**6.35**	**8.27**	**4.43**
FLOT1	flotillin 1	6p21.3	2.63	2.71	2.56
ELF3	E74-like factor 3 (ets domain transcription factor, epithelial-specific)	1q32.2	7.49	7.98	6.99
CD151	CD151 antigen	11p15.5	11.53	13.49	9.57
SULT1A4	Sulfotransferase family, cytosolic, 1A, phenol-preferring, member 4	16p11.2	2.07	1.99	2.15
PPIB	peptidylprolyl isomerase B (cyclophilin B)	15q21-q22	2.35	2.34	2.35
SLC12A7	solute carrier family 12 (potassium/chloride transporters), member 7	5p15	2.10	2.03	2.17
AARS	alanyl-tRNA synthetase	16q22	2.28	2.13	2.44
SEZ6L2	Seizure related 6 homolog (mouse)-like 2	16p11.2	3.23	3.03	3.44
GABARAPL1	GABA(A) receptor-associated protein like 1	12p13.31	2.31	2.25	2.37
ATP9A	ATPase, Class II, type 9A	20q13.11-13.2	2.56	2.86	2.25
PIM1	pim-1 oncogene	6p21.2	2.05	2.25	1.84
TNP1	transition protein 1 (during histone to protamine replacement)	16p13.13	2.03	2.18	1.88
DPP7	dipeptidylpeptidase 7	9q34.3	2.59	2.56	2.61
SPOCK1	sparc/osteonectin, cwcv and kazal-like domains proteoglycan (testican) 1	5q31	2.07	1.70	2.44
FOXM1	forkhead box M1	12p13	2.52	2.47	2.57
PMS2	PMS2 postmeiotic segregation increased 2 (S. cerevisiae)	7p22	2.05	1.78	2.31
SLC27A2	fatty-acid-Coenzyme A ligase, very long-chain 1	15q21.2	1.98	2.42	1.54
GATA2	GATA binding protein 2	3q21	2.48	2.88	2.07
EPB41L4B	EHM2 gene	9q22.1-q22.3	2.04	1.91	2.17
BLMH	bleomycin hydrolase	17q11.2	2.13	2.29	1.96
CYBA	cytochrome b-245, alpha polypeptide	16q24	10.55	14.32	6.78
CTSH	cathepsin H	15q24-q25	4.55	5.42	3.68
ALDH1A3	aldehyde dehydrogenase 1 family, member A3	15q26	2.65	2.90	2.39
KHK	ketohexokinase (fructokinase)	2p23.3-p23.2	2.15	2.56	1.75
LCN2	lipocalin 2 (oncogene 24p3)	9q34	2.97	3.30	2.65
KLRC2	killer cell lectin-like receptor subfamily C, member 2	12p13	2.04	2.82	1.25
CED-6	CED-6 protein	2q32.3-q33	2.25	2.56	1.93

Shown are gene list and fold change in cluster C over-expressed in Head and Neck Squamous Cell Carcinoma (HNSCC).
[1] Order of gene refers to the order in the tree. Shown in bold are three subclusters C1, C2 and C3 of cluster C.
[2] fold change = average of 10 HNSCC vs. average of 4 Human Normal Keratinacytes (HKC)
[3] fold change = average of 5 HNSCC of wild type (wt) p53-like vs. average of 4 HKC
[4] fold change = average of 5 HNSCC of mutant (mt) p53 vs. average of 4 HKC
