# Supplementary material for: Genome-wide identification of novel expression signatures reveal distinct patterns and prevalence of binding motifs for p53, nuclear factor-κB and other signal transcription factors in head and neck squamous cell carcinoma
Source: Genome Biol. 2007 May 11;8(5):R78. doi: 10.1186/gb-2007-8-5-r78 (PMC1929156; doi:10.1186/gb-2007-8-5-r78)
Supplement: Additional data file 2 — Included are GO annotations of genes in clusters A to C (over-expressed in UM-SCC cells) and of genes in clusters D to F (under-expressed in UM-SCC cells). [file gb-2007-8-5-r78-S2.pdf]

Supplemental Table S2. Gene Ontology Annotation of HNSCC clusters

Cluster	Number[1]	%[2]	Gene Ontology Annotation	*P* value[3]
Up-regulated:				
A	8	23.5	nucleosome assembly	1.40×10^{-12}
	7	20.6	chromosome organization and biogenesis (sensu Eukaryota)	8.82×10^{-10}
	9	26.5	DNA binding	1.19×10^{-4}
	3	8.8	transport	1.65×10^{-2}
	3	8.8	calcium ion binding	3.53×10^{-2}
B	3	8.1	negative regulation of cell proliferation	1.60×10^{-3}
	4	10.8	cell-cell signaling	1.60×10^{-3}
	3	8.1	cell surface receptor linked signal transduction	2.00×10^{-3}
	9	24.3	protein binding	4.80×10^{-3}
	4	10.8	DNA binding	3.80×10^{-2}
C	10	4.2	DNA replication	2.53×10^{-4}
	8	3.3	ubiquitin cycle	3.86×10^{-2}
	6	2.5	cell division	4.40×10^{-2}
	9	3.8	catalytic activity	5.10×10^{-3}
	15	6.3	oxidoreductase activity	2.20×10^{-2}
C1	3	6.7	lipid metabolism	7.09×10-3
	3	6.7	inflammatory response	3.84×10-3
	4	8.8	oxidoreductase activity	1.34×10-2
	4	8.8	calcium ion binding	2.53×10-2
	7	15.6	regulation of transcription, DNA-dependent	2.47×10-2
C2	4	10.3	mitosis	3.45×10-5
	4	10.3	DNA replication	4.87×10-5
	4	10.3	regulation of cell cycle	4.21×10-4
	8	20.5	ATP binding	1.16×10-3
	4	10.3	nucleotide binding	1.34×10-3
C3	2	4.0	intracellular transporter activity	3.50×10-4
	2	4.0	phosphoinositide-mediated signaling	7.61×10-4
	3	6.0	DNA replication	5.67×10-3
	3	6.0	DNA repair	6.50×10-3
	3	6.0	binding	2.59×10-2
Down-regulated:				
D	14	5.6	epidermis development	1.40×10^{-12}
	17	6.8	structural molecule activity	2.85×10^{-6}
	9	3.6	structural constituent of cytoskeleton	2.91×10^{-4}
	14	5.6	cell adhesion	7.20×10^{-3}
	11	4.4	cell-cell signaling	1.00×10^{-2}
	10	4.0	cell differentiation	1.10×10^{-2}
	10	4.0	immune response	2.90×10^{-2}
	18	7.2	calcium ion binding	2.60×10^{-2}
	16	6.4	regulation of transcription, DNA-dependent	4.90×10^{-2}
E	21	10.0	calcium ion binding	4.58×10^{-4}
	9	4.3	actin binding	7.60×10^{-3}
	29	13.7	signal transduction	1.30×10^{-2}
	11	5.2	cell proliferation	2.40×10^{-2}
	8	3.8	G-protein coupled receptor protein signaling pathway	3.00×10^{-2}

	9	4.3	regulation of cell cycle	3.80×10^{-2}
	9	4.3	signal transducer activity	4.00×10^{-2}
F	6	3.1	heparin binding	8.28×10^{-4}
	7	3.6	morphogenesis	2.90×10^{-3}
	6	3.1	serine-type endopeptidase inhibitor activity	2.40×10^{-3}
	4	2.0	damaged DNA binding	5.50×10^{-3}
	6	3.1	positive regulation of cell proliferation	7.10×10^{-3}
	11	5.6	cell adhesion	1.90×10^{-2}
	8	4.1	cell differentiation	2.30×10^{-2}
	5	2.6	DNA binding	4.52×10^{-2}

Shown are Gene Ontology (GO) annotation in up-regulated cluster A-C and down-regulated D-F in Head and Neck Squamous Cell Carcinoma (HNSCC). C1, C2 and C3 were three subclusters in cluster C. Genes were mapped to GO Biological process and Molecular function by using Onto-Express (Draghici et al. 2003). [2] refer to gene number for a mapped functional GO category within the entire cluster. [3] refer to percentage of gene within the entire cluster for a mapped functional GO category. [4] indicate corrected *P* value for statistical significant enrichment.
