# Supplementary material for: Genome-wide identification of novel expression signatures reveal distinct patterns and prevalence of binding motifs for p53, nuclear factor-κB and other signal transcription factors in head and neck squamous cell carcinoma
Source: Genome Biol. 2007 May 11;8(5):R78. doi: 10.1186/gb-2007-8-5-r78 (PMC1929156; doi:10.1186/gb-2007-8-5-r78)
Supplement: Additional data file 3 — Provided are sequences, location, and matrix similarity of putative TFBSs in genes in clusters A and B (over-expressed in UM-SCC cells). [file gb-2007-8-5-r78-S3.pdf]

Supplemental Table S3. Putative transcription factor binding sites of clusterA and B over-repressed in HNSCC

Cluster A:

Symbol	Gene description	Refseq	TF Family[1]	TF Matrix [2]	Start position [3]	Strand	Matrix similiarity [4]	Core similiarity [4]	TF binding sequence
ABCC5	ATP-binding cassette, sub-family C (CFTR/MRP), member 5	NM_005688	V$STAT	STAT3.01	152	+	0.83	0.75	ggcgcTTCTgggaaatgta
					152	-	0.831	1	tacatTTCCcagaagcgcc
ARID1A	AT rich interactive domain 1A (SWI- like)	NM_139135	V$EGR	EGR1.01	445	-	0.792	1	agccgcggcGGCGgcag
				EGR1.02	486	+	0.869	0.789	gccttcggCGGCggcgc
					483	+	0.865	0.789	ttcggcggCGGCgctgc
					445	-	0.871	0.789	agccgcggCGGCggcag
					134	-	0.862	1	agcagccgGGGCgccgg
					59	-	0.973	1	gggggaggGGGCgggga
					-45	-	0.865	1	aatgagggGGGCggtgg
			V$p53	p53.01	-54	-	0.665	0.75	gccCTTGcctgggaatgaggg
ARTS-1	type 1 tumor necrosis factor receptor shedding aminopeptidase regulator	NM_016442	V$EGR	EGR1.02	-35	+	0.867	0.789	ggtggtggCGGCggcgc
			V$NFκB	CREL.01	82	+	0.923	1	ccaggggcTTCCccg
				NFKAPPAB.01	81	+	0.951	0.904	caGGGGcttccccgc
				NFKAPPAB.02	81	+	0.825	0.75	caGGGGcttccccgc
				NFKAPPAB.03	82	-	0.869	1	cgGGGAagcccctgg
				NFKAPPAB50.01	82	-	0.849	0.8	cgGGGAagcccctgg
					81	-	0.892	1	gcGGGGaagcccctg
					81	+	0.921	1	caGGGGcttccccgc
				NFKAPPAB65.01	81	+	0.898	0.826	caggggctTCCCcgc
			V$p53	p53.03	447	-	0.94	0.922	aggcaggcgctgaCAAGtcca
				p53.01	446	+	0.693	0.75	ggaCTTGtcagcgcctgcctg
			V$STAT	STAT3.01	156	-	0.764	0.75	gcaacTCCCaggaagggaa
ATP1B3	ATPase, Na+/K+ transporting, beta 3 polypeptide	NM_001679	V$EGR	EGR1.01	305	-	0.801	0.831	ggttgcttaGGAGgagg
					144	-	0.804	1	ggcggcgagGGCGcccc
					-88	-	0.856	1	ggctgcggaGGCGcgtc
				EGR1.02	344	-	0.863	1	gggcgccgGGGCgctga
					161	+	0.916	1	cggggccgGGGCggggt
					138	-	0.89	0.789	gcctgaggCGGCgaggg
					123	-	0.895	1	agcggccgGGGCgctgc
					-88	-	0.9	0.842	ggctgcggAGGCgcgtc
			V$NFκB	CREL.01	329	-	0.943	1	cctgggcgTTCCtgg
				NFKAPPAB.01	189	-	0.883	0.904	tcGGGGgctccctca
				NFKAPPAB50.01	282	+	0.892	1	gcGGGGacgcccgcc
					189	-	0.838	1	tcGGGGgctccctca
BLK	B lymphoid tyrosine kinase	NM_001715	V$AP1	AP1FJ.01	482	+	0.971	1	agTGACttcct
CDC42EP4	CDC42 effector protein (Rho GTPase binding) 4	NM_012121	V$NFκB	NFKAPPAB.02	81	-	0.823	1	agGGGActgtcattt
				NFKAPPAB65.01	352	+	0.879	0.783	gctggattTACCttc
			V$p53	p53.03	-63	-	0.921	0.828	ccgaggtagccggCAGGtctg
			V$STAT	STAT3.01	368	+	0.757	0.75	cttgcTGCCtggaattgct
					368	-	0.743	1	agcaaTTCCaggcagcaag
					243	+	0.769	0.75	caagtTTCAgggaaagtgt
					243	-	0.758	1	acactTTCCctgaaacttg
CDKN2C	cyclin-dependent kinase inhibitor 2C (p18)	NM_078626	V$NFκB	HIVEP1.01	146	-	0.861	1	aaGGGAtagtcctgg
			V$p53	p53.01	99	+	0.69	0.75	ggaCCTGcccctgcagttctg
				p53.02	109	+	0.919	0.82	gccccctggaggaCCTGcccc

				p53.03	429	-	0.933	0.828	agcgctagcggggCAGGtcgg
					100	-	0.931	0.828	agaactgcaggggCAGGtcct
CKB	creatine kinase, brain	NM_001823	V$EGR	EGR1.01	373	-	0.792	1	ccccgcggcGGCGgcga
					157	+	0.803	1	gctcgggtgGGCGgcca
					-38	-	0.816	1	gggggcgggGGCGctcc
					-44	-	0.846	1	ccgggcgggGGCGgggg
				EGR1.02	432	+	0.903	1	cgggggtgGGGCggggg
					426	+	0.879	1	tggggcggGGGCcgtac
					376	-	0.901	0.789	cgcggcggCGGCgaggg
					373	-	0.887	0.789	ccccgcggCGGCggcga
					366	+	0.874	1	cgccgcggGGGCtggct
					-38	-	0.93	1	gggggcggGGGCgctcc
					-44	-	0.978	1	ccgggcggGGGCggggg
					-81	+	0.879	1	ggggccggGGGCgtccg
			V$NFκB	CREL.01	493	-	0.933	1	gcaggcgtTTCCtgg
					405	-	0.943	1	ttcggggcTTCCgca
				NFKAPPAB.01	-19	+	0.883	1	ccGGGAgctgccgac
				NFKAPPAB65.01	493	-	0.904	1	gcaggcgtTTCCtgg
CPS1	carbamoyl-phosphate synthetase 1, mitochondrial	NM_001875	V$AP1	AP1.02	5	-	0.961	1	gcTGACtgcac
			V$NFκB	CREL.01	231	+	0.919	1	ctaggtcaTTCCatt
			V$p53	p53.01	106	-	0.691	0.75	ggtAATGtccagagatgttcc
					105	+	0.697	0.75	gaaCATCtctggacattacct
				p53.02	128	+	0.911	1	gcaatttgtgttaCATGccca
FZD1	frizzled homolog 1 (Drosophila)	NM_003505	V$EGR	EGR1.01	339	-	0.798	0.831	ggatacgtaGGAGaatg
					-23	+	0.81	1	tcaggcggcGGCGgcgg
					-26	+	0.794	1	ggcggcggcGGCGgaga
					-44	+	0.804	1	ggaggcggaGGCGcagg
				EGR1.02	219	-	0.884	0.842	gtaggaggAGGCgggag
					-23	+	0.867	0.789	tcaggcggCGGCggcgg
					-26	+	0.92	0.789	ggcggcggCGGCggaga
					-44	+	0.882	0.842	ggaggcggAGGCgcagg
			V$NFκB	NFKAPPAB.02	397	-	0.844	1	gcGGGActtcgcttt
HARSL	histidyl-tRNA synthetase-like	NM_012208	V$EGR	EGR1.01	444	-	0.817	0.831	aatggcgggGGAGggag
			V$NFκB	CREL.01	-13	-	0.92	1	ttcggcacTTCCggg
				HIVEP1.01	-46	-	0.836	1	gtGGGAtcctcccaa
				NFKAPPAB.03	-46	-	0.857	1	gtGGGAtcctcccaa
			V$STAT	STAT3.01	-6	+	0.801	0.75	ggctcCTCCcggaagtgcc
					-6	-	0.8	1	ggcacTTCCgggaggagcc
HBE1	hemoglobin, epsilon 1	NM_005330	V$NFκB	CREL.01	417	-	0.947	1	tggggtgaTTCCcta
				HIVEP1.01	417	+	0.856	1	taGGGAatcacccca
				NFKAPPAB.02	417	+	0.857	1	taGGGAatcacccca
				NFKAPPAB.03	417	+	0.87	1	taGGGAatcacccca
			V$STAT	STAT3.01	109	+	0.765	0.75	aggctTTCTtggaaaagga
					109	-	0.748	1	tcctTTCCaagaaagcct
HIST1H2AC	H2A histone family, member L	NM_003512	V$NFκB	NFKAPPAB.02	167	-	0.821	0.75	gaGGTActctccatc
HIST1H2AM	H2A histone family, member N	NM_003514	V$p53	p53.01	238	-	0.684	0.75	gggCATAacccagcttgtcct
					237	+	0.673	0.75	ggaCAAGctgggttatgccct
					-31	-	0.672	0.75	tgcCACGtccagacatggtaa
					-32	+	0.678	1	tacCATGtctggacgtggcaa
				p53.02	284	-	0.931	1	cactctcctccgcCATGcccg
HIST1H2BC	H2B histone family, member L	NM_003526	V$NFκB	NFKAPPAB.02	88	+	0.821	0.75	gaGGTActctccatc
HIST1H2BD	H2B histone family, member B	NM_138720	V$NFκB	HIVEP1.01	402	-	0.925	1	ttGGGAcatcccccc

					401	+	0.877	1	ggGGGAtgtcccaat
				NFKAPPAB.01	402	-	0.947	1	ttGGGAcatcccccc
					401	+	0.927	1	ggGGGAtgtcccaat
				NFKAPPAB.02	402	-	0.853	1	ttGGGAcatcccccc
					401	+	0.831	1	ggGGGAtgtcccaat
				NFKAPPAB.03	402	-	0.902	1	ttGGGAcatcccccc
					401	+	0.901	1	ggGGGAtgtcccaat
				NFKAPPAB50.01	402	+	0.891	1	ggGGGGatgtcccaa
			V$p53	p53.03	439	-	0.949	0.922	ttttacgtttcggCAAGtcct
HIST1H2BJ	H2B histone family, member R	NM_021058	V$EGR	EGR1.01	-13	+	0.81	1	cttttcgttGGCGcttt
			V$STAT	STAT3.01	475	+	0.779	1	gccagTTCCaggatctcgg
					475	-	0.782	0.75	ccgagATCCtggaactggc
HIST1H2BL	H2B histone family, member C	NM_003519	V$p53	p53.01	295	-	0.731	1	tgcCATGtctgggcgtggcaa
					294	+	0.716	0.75	tgcCACGcccagacatggcaa
HIST1H2BN	H2B histone family, member D	NM_003520	V$p53	p53.01	339	-	0.694	1	cgtCATGtcgggacgtggcaa
					338	+	0.696	0.75	tgcCACGtcccgacatgacgt
				p53.02	14	+	0.96	1	agttactcccagtCATGcccg
			V$STAT	STAT3.01	145	-	0.761	0.75	ggtgaGTCCtggaaacgta
					27	+	0.745	0.75	ccaatTTTCcggcagttac
					27	-	0.74	0.75	gtaacTGCCggaaaattgg
HIST2H2BE	H2B histone family, member Q	NM_003528	V$EGR	EGR1.02	434	-	0.874	1	gcgggtggGGGCcggcg
			V$NFκB	NFKAPPAB.01	328	+	0.884	0.815	gcGGGCcttgcctcc
			V$p53	p53.01	309	-	0.698	1	agtCATGtctggtcgtggcaa
					308	+	0.692	0.75	tgcCACGaccagacatgactg
					23	-	0.666	0.75	gagAATGggcgggcctgattc
				p53.03	299	-	0.932	1	ccttgatttcagtCATGtctg
			V$STAT	STAT3.01	381	+	0.776	0.75	cgcccTACCgggaactgga
					381	-	0.78	1	tccagTTCCcggtagggcg
IGFBP2	insulin-like growth factor binding protein 2, 36kDa	NM_000597	V$EGR	EGR1.01	96	+	0.799	0.831	cggcgcgagGGAGtgtc
					3	+	0.832	1	ggctgcggcGGCGaggg
					-3	+	0.79	0.831	ggcggcgagGGAGgagg
				EGR1.02	41	+	0.887	1	aaggcaggGGGCgggga
					3	+	0.895	0.789	ggctgcggCGGCgaggg
					-24	+	0.91	0.842	agcggaggAGGCggctc
			V$NFκB	CREL.01	162	-	0.939	1	tctgggggTTCCctg
				NFKAPPAB.01	163	-	0.934	0.904	ctGGGGgttccctgc
				NFKAPPAB50.01	163	-	0.915	1	ctGGGGgttccctgc
					162	+	0.833	0.8	caGGGAacccccaga
				NFKAPPAB65.01	163	-	0.898	0.826	ctgggggTCCCtgc
LGALS3BP	lectin, galactoside-binding, soluble, 3 binding protein	NM_005567	V$NFκB	CREL.01	187	+	0.957	1	ggcggcctTTCCaac
				NFKAPPAB65.01	187	+	0.916	1	ggcggcctTTCCaac
			V$STAT	STAT3.01	13	+	0.774	0.75	ggtggTTTCtggaaatcga
					13	-	0.767	1	tcgatTTCCagaaaccacc
					1	+	0.766	1	agcatTTCCtgggatcagc
					1	-	0.798	0.75	gctgaTCCCaggaaatgct
MATN2	matrilin 2	NM_030583	V$p53	p53.02	116	+	0.921	1	aattccaactatgCATGccct
MYST3	MYST histone acetyltransferase (monocytic leukemia) 3	NM_006766	V$EGR	EGR1.01	177	-	0.79	0.826	ggcagcgcgGGTGgggg
					140	-	0.79	1	aagagcctgGGCGggcg
				EGR1.02	238	-	0.898	1	ggagggcgGGGCggggc
					184	-	0.912	1	cgggtgggGGGCggccg
					157	-	0.885	0.842	gcgaggggAGGCggagg
					144	-	0.876	1	gcctgggcGGGCggcga

					-82	-	0.89	1	gcggccggGGGCggccg
			V$NFκB	CREL.01	347	-	0.943	1	agcgggggTTCCtcc
					255	+	0.914	1	gccggctcTTCCctg
					224	+	0.957	1	tccggcctTTCCcgg
					74	+	0.914	1	cgcggctgTTCCgcc
					-70	+	0.93	1	aggggccgTTCCcgg
				NFKAPPAB65.01	224	+	0.916	1	tccggcctTTCCcgg
OLFM1	olfactomedin 1	NM_058199	V$EGR	EGR1.01	8	-	0.801	0.826	gggggcgggGGTGgcgc
					2	-	0.846	1	aggggcgggGGCGgggg
				EGR1.02	11	-	0.889	0.789	ggcgggggTGGCgcaga
					2	-	0.966	1	aggggcggGGGCggggg
					-4	-	0.919	1	ctcggaagGGGCggggg
					-243	-	0.882	1	cccggagcGGGCggcgc
			V$NFκB	NFKAPPAB50.01	194	-	0.89	1	gaGGGGttgcccgcg
			V$p53	p53.01	-153	-	0.703	0.75	gggCAGGgctgggcagggctg
					-154	+	0.671	0.75	agcCCTGcccagccctgcccg
			V$STAT	STAT3.01	391	-	0.773	1	gatgtTTCCaggaggaggg
					175	+	0.768	0.75	cccatTCCCgggacctgga
					175	-	0.752	0.75	tccagGTCCcgggaatggg
SLC9A3R1	solute carrier family 9, isoform 3 regulatory factor 1	NM_004252	V$EGR	EGR1.02	247	-	0.897	1	ggtggcagGGGCggggc
					45	-	0.887	1	taccccggGGGCggggc
					-3	+	0.867	1	gccgcgcgGGGCgggga
			V$NFκB	NFKAPPAB50.01	70	-	0.833	1	gtGGGGacctccgcg
TDRD7	tudor domain containing 7	NM_014290	V$EGR	EGR1.01	21	-	0.82	1	gcacgcgcgGGCGctcc
				EGR1.02	208	-	0.87	1	tcagcaggGGGCgaggg
					136	-	0.866	1	cgcctgcgGGGCgggtg
					-24	+	0.899	1	gcggggcgGGGCgaagc
					-54	+	0.898	1	gcagggcgGGGCggggg
					-60	+	0.882	1	cggggcggGGGCttgag
			V$NFκB	CREL.01	-73	+	0.915	1	tgaggtgaTTCCcaa
				HIVEP1.01	-73	-	0.859	1	ttGGGAatcacctca
				NFKAPPAB.02	-73	-	0.854	1	ttGGGAatcacctca
				NFKAPPAB.03	-73	-	0.875	1	ttGGGAatcacctca
			V$p53	p53.03	399	+	0.933	1	ggaaggaggttgaCATGttag
TGM1	transglutaminase 1	NM_000359	V$AP1	AP1.02	43	-	0.981	1	agTGACttatg
				AP1.03	43	-	0.984	1	agTGACttatg
				AP1FJ.01	43	-	0.985	1	agTGACttatg
			V$EGR	EGR1.02	80	-	0.92	1	gctgtaggGGGCggaga
			V$p53	p53.01	452	-	0.662	0.75	catCCTGcccagggaggtcct
					230	+	0.676	1	tcaCATGcccagtctggtagg
				p53.02	240	+	0.924	1	aaggggctcctcaCATGccca
THAP11	THAP domain containing 11	NM_020457	V$EGR	EGR1.01	-21	+	0.806	1	ccgagcgcaGGCGggca
				EGR1.02	76	-	0.878	1	cacttccgGGGCgggtg
UBADC1	ubiquitin associated domain containing 1	NM_016172	V$EGR	EGR1.01	17	+	0.862	1	tgctgcggcGGCGggag
					-78	+	0.833	1	gagcgcgcgGGCGgtct
					-159	+	0.862	1	ccctgcggcGGCGgcgg
					-162	+	0.794	1	tgcggcggcGGCGggat
				EGR1.02	133	-	0.889	1	gccgccggGGGCgcgct
					34	-	0.889	1	gccgccggGGGCgcgcc
					17	+	0.895	0.789	tgctgcggCGGCgggag
					-46	+	0.886	1	gggagccgGGGCggggc

					-51	+	0.905	1	ccggggcgGGGCggcgg
					-96	-	0.973	1	cggggaggGGGCggggc
					-159	+	0.905	0.789	ccctgcggCGGCggcgg
					-162	+	0.901	0.789	tgcggcggCGGCgggat
			V$p53	p53.01	193	-	0.688	1	gagCATGccgggagctgtagt
					93	-	0.739	1	gcgCATGccgggagatgtggt
					92	+	0.728	0.75	ccaCATCtcccggcatgcgcc
XCL1	chemokine (C motif) ligand 1	NM_002995	V$p53	p53.02	332	+	0.914	1	aacagccttcaggCATGtcta
					323	-	0.922	1	cttccaccgtagaCATGcctg
				p53.03	332	+	0.991	1	aacagccttcaggCATGtcta
XPA	xeroderma pigmentosum, complementation group A	NM_000380	V$AP1	AP1.01	237	+	0.953	0.885	tctgaATCAcc
					237	-	0.957	0.846	ggtgaTTCAga
			V$EGR	EGR1.01	69	+	0.845	1	aactgcgcaGGCGctct
				EGR1.02	-88	+	0.901	0.789	gccggaggCGGCggctt
			V$p53	p53.01	131	-	0.661	0.75	gggTATGcgcggacacggagt
				p53.03	314	-	0.928	0.922	gtgtgaccttgggCAAGttat

Cluster B:

Symbol	Gene description	Refseq	TF Family[1]	TF Matrix [2]	tart position	Strand	Matrix similia	Core similarit	TF binding sequence
ABCG2	ATP-binding cassette, sub-family G (WHITE), member 2	NM_004827	V$AP1	AP1.01	-78	-	0.99	1.00	gatgaGTCAcc
					-78	+	0.97	0.94	ggtgaCTCAtc
				AP1.02	-78	+	1.00	1.00	ggTGACtcatc
				AP1.03	-78	+	1.00	1.00	ggTGACtcatc
				AP1FJ.01	-78	+	1.00	1.00	ggTGACtcatc
			V$EGR	EGR1.01	394	-	0.81	1.00	gggttcgcgGGCGgggg
					204	-	0.86	0.83	gccggcgtgGGAGgcgc
				EGR1.02	207	-	0.87	0.84	ggcgtgggAGGCgctgc
			V$NFκB	CREL.01	-65	-	0.93	1.00	cccggaccTTCCaaa
				NFKAPPAB50.01	373	+	0.89	1.00	ctGGGGaaacccggg
					258	-	0.90	1.00	ctGGGGagacccgga
					373	-	0.88	0.83	cccgggttTCCCcag
ACSL5	acyl-CoA synthetase long-chain family member 5	NM_016234	V$EGR	EGR1.02	221	+	0.87	1.00	atctgaggGGGCacctc
			V$NFκB	NFKAPPAB.01	-8	-	0.88	1.00	cgGGGActtcacttc
				NFKAPPAB.02	-8	-	0.85	1.00	cgGGGActtcacttc
				NFKAPPAB.03	-8	-	0.85	1.00	cgGGGActtcacttc
AKAP12	A kinase (PRKA) anchor protein (gravin) 12	NM_005100	V$EGR	EGR1.02	178	+	0.86	1.00	ggccgggtGGGCggctg
					47	+	0.91	1.00	ggggagggGGGCggagg
			V$NFκB	CREL.01	61	-	0.96	1.00	cccggtttTTCCctc
				NFKAPPAB.03	232	-	0.84	1.00	cgGGGAcgacccagg
				NFKAPPAB65.01	61	-	0.91	1.00	cccggtttTTCCctc
			V$p53	p53.01	95	+	0.66	0.75	gggCGTGtctggggctgtgct
ARID3A	AT rich interactive domain 3A (BRIGHT- like)	NM_005224	V$EGR	EGR1.01	-35	-	0.85	1.00	gggggcgggGGCGgggg
					-80	-	0.82	0.80	tgcagcgtgGGGGccgc
				EGR1.02	-29	-	0.87	1.00	gggggcggGGGCcgccg
					-35	-	0.98	1.00	gggggcggGGGCggggg
					-41	-	0.90	1.00	gtggcaggGGGCggggg
			V$NFκB	CREL.01	359	-	0.94	1.00	aatgggcgTTCCtgg
			V$p53	p53.02	12	+	0.96	1.00	cgtgaggaagaggCATGcccc
					3	-	0.94	1.00	aaggctgatggggCATGcctc
BCAT1	branched chain aminotransferase 1, cytosolic	NM_005504	V$AP1	AP1.02	157	+	0.99	1.00	ggTGACtaagg
				AP1.03	157	+	1.00	1.00	ggTGACtaagg

				AP1FJ.01	157	+	0.99	1.00	ggTGACtaagg
			V$EGR	EGR1.02	-5	-	0.86	0.79	cgaggcggCGGCgagta
					-8	-	0.88	0.79	gcccgaggCGGCggcga
					-105	+	0.87	1.00	ccatctggGGGCggcct
			V$NFκB	NFKAPPAB50.01	-100	-	0.97	1.00	gtGGGGatgcccgtg
					-101	+	0.90	0.77	acGGGCatccccaca
BIRC2	baculoviral IAP repeat-containing 2	NM_001166	V$AP1	AP1.02	496	+	0.97	1.00	acTGACtccat
					408	+	0.96	1.00	ggTGACacagg
				AP1FJ.01	408	+	0.98	1.00	ggTGACacagg
			V$EGR	EGR1.01	75	-	0.80	1.00	ccgcgcgagGGCGcccg
				EGR1.02	3	+	0.92	1.00	cccggatgGGGCggcgg
			V$NFκB	CREL.01	372	+	0.97	1.00	cagggcgtTTCCgca
				HIVEP1.01	228	-	0.90	1.00	cgGGGActatccgta
				NFKAPPAB.01	372	+	0.91	0.82	caGGGCgtttccgca
					228	-	0.90	1.00	cgGGGActatccgta
				NFKAPPAB.02	372	+	0.83	0.75	caGGGCgtttccgca
					228	-	0.93	1.00	cgGGGActatccgta
				NFKAPPAB.03	228	-	0.91	1.00	cgGGGActatccgta
				NFKAPPAB65.01	372	+	0.93	1.00	cagggcgtTTCCgca
			V$STAT	STAT3.01	145	+	0.80	1.00	cgtctTTCCcggaggttca
					145	-	0.77	0.75	tgaacCTCCgggaaagacg
CA9	carbonic anhydrase IX	NM_001216	V$AP1	AP1.01	59	+	1.00	1.00	tgtgaGTCAgc
					59	-	0.97	0.94	gctgaCTCAca
				AP1.02	59	-	1.00	1.00	gcTGACtcaca
				AP1.03	59	-	0.98	1.00	gcTGACtcaca
				AP1FJ.01	59	-	0.98	1.00	gcTGACtcaca
			V$NFκB	HIVEP1.01	-28	+	0.84	1.00	ctGGGAcaccccaca
				NFKAPPAB.01	-27	-	0.89	0.90	gtGGGGtgtcccagc
				NFKAPPAB50.01	-27	-	0.86	1.00	gtGGGGtgtcccagc
CAP2	CAP, adenylate cyclase-associated protein, 2	NM_006366	V$AP1	AP1.03	-108	+	0.96	1.00	cgTGACtgaca
CROC4	transcriptional activator of the c-fos promoter	NM_006365	V$p53	p53.02	210	+	0.92	1.00	ccaggccggaggcCATGcctg
DMAP1	DNA methyltransferase 1 associated protein 1	NM_019100	V$AP1	AP1FJ.01	249	+	0.97	1.00	agTGACttccc
			V$NFκB	CREL.01	-75	-	0.92	1.00	cttggatgTTCCtaa
				HIVEP1.01	250	+	0.84	0.75	aaGTGActtcccaaa
				NFKAPPAB.02	250	+	0.83	0.75	aaGTGActtcccaaa
				NFKAPPAB.02	183	-	0.84	0.75	ctGGCAttttccctc
DNAH11	dynein, axonemal, heavy polypeptide 11	NM_003777	V$EGR	EGR1.01	58	+	0.85	0.83	gcctgcggaGGTGtcct
				EGR1.02	260	+	0.86	0.79	ctgggcggTGGCgcggt
					237	-	0.93	1.00	cccgctggGGGCggagt
					225	+	0.91	1.00	gcgggctgGGGCgggga
FADS3	fatty acid desaturase 3	NM_021727	V$EGR	EGR1.01	-49	+	0.79	1.00	gcgggcggcGGCGgagg
					-55	+	0.82	1.00	ggcggcggaGGCGgcgc
					-147	+	0.79	1.00	tgcagcatgGGCGgcgt
				EGR1.02	259	+	0.86	1.00	ggaaaaggGGGCgggac
					204	+	0.91	1.00	cccgggagGGGCgggcc
					90	+	0.90	1.00	ctggccggGGGCggggg
					82	+	0.87	1.00	gggcggggGGGCaggcg
					-49	+	0.90	0.79	gcgggcggCGGCggagg
					-55	+	0.93	0.84	ggcggcggAGGCggcgc
					-58	+	0.86	0.79	ggcggaggCGGCgcccg
			V$NFκB	CREL.01	415	-	0.96	1.00	tggggggcTTCCagc
					32	+	0.96	1.00	gcggggagTTCCctg

				HIVEP1.01	32	-	0.87	1.00	caGGGAactccccgc
					31	+	0.92	1.00	cgGGGAgttccctgc
				NFKAPPAB.01	415	-	0.89	0.90	tgGGGGgcttccagc
					32	-	0.95	1.00	caGGGAactccccgc
					32	+	0.92	0.90	gcGGGGagttccctg
					31	+	0.97	1.00	cgGGGAgttccctgc
				NFKAPPAB.02	32	-	0.82	1.00	caGGGAactccccgc
					31	+	0.92	1.00	cgGGGAgttccctgc
				NFKAPPAB.03	32	-	0.89	1.00	caGGGAactccccgc
					31	+	0.93	1.00	cgGGGAgttccctgc
				NFKAPPAB50.01	32	+	0.86	1.00	gcGGGGagttccctg
					32	-	0.84	0.80	caGGGAactccccgc
					31	+	0.84	0.80	cgGGGAgttccctgc
					-122	-	0.88	1.00	ctGGGGatcccaggc
					32	+	0.88	1.00	gcggggagTTCCctg
					31	+	0.92	0.83	cggggagtTCCCtgc
ICAM1	intercellular adhesion molecule 1 (CD54)	NM_000201	V$AP1	AP1.01	269	-	0.95	0.89	cttgaATCAcg
			V$EGR	EGR1.01	469	-	0.81	0.83	ccctgcctgGGTGgggg
					396	-	0.86	0.80	ggctgcgagGGGGtccg
					148	+	0.79	0.83	gccagcgagGGAGgatg
			V$NFκB	CREL.01	172	+	0.95	1.00	cttggaaaTTCCgga
					172	-	0.97	1.00	tccggaatTTCCaag
				HIVEP1.01	172	-	0.85	0.75	tcCGGAatttccaag
				NFKAPPAB.01	-117	+	0.93	0.90	gtGGGGattgccgtc
				NFKAPPAB.02	172	-	0.85	0.75	tcCGGAatttccaag
				NFKAPPAB.03	172	+	0.85	0.82	ctTGGAaattccgga
					172	-	0.86	0.78	tcCGGAatttccaag
				NFKAPPAB50.01	-117	+	0.91	1.00	gtGGGGattgccgtc
				NFKAPPAB65.01	172	-	0.98	1.00	tccggaatTTCCaag
					172	+	0.90	1.00	cttggaaaTTCCgga
					-117	+	0.90	0.78	gtggggatTGCCgtc
			V$STAT	STAT3.01	63	+	0.89	1.00	gaggtTTCCgggaaagcag
					63	-	0.87	1.00	ctgctTTCCcggaaacctc
IL6	interleukin 6 (interferon, beta 2)	NM_000600	V$AP1	AP1.01	286	+	0.98	1.00	gctgaGTCAct
					286	-	0.98	0.94	agtgaCTCAgc
				AP1.02	286	-	0.99	1.00	agTGACtcagc
				AP1.03	286	-	1.00	1.00	agTGACtcagc
				AP1FJ.01	286	-	0.99	1.00	agTGACtcagc
			V$EGR	EGR1.01	497	+	0.82	0.83	ggctgcgatGGAGtcag
			V$NFκB	CREL.01	76	+	0.98	1.00	gtgggattTTCCcat
				HIVEP1.01	76	-	0.87	1.00	atGGGAaaatcccac
					76	+	0.96	1.00	gtGGGAttttcccat
				NFKAPPAB.01	76	+	0.98	1.00	gtGGGAttttcccat
				NFKAPPAB.02	76	+	0.96	1.00	gtGGGAttttcccat
				NFKAPPAB.03	76	+	0.95	1.00	gtGGGAttttcccat
					76	-	0.86	1.00	atGGGAaaatcccac
				NFKAPPAB65.01	76	+	0.98	1.00	gtgggattTTCCcat
			V$p53	p53.02	301	-	0.91	1.00	ctcagcactttggCATGtctt
				p53.03	301	-	0.96	1.00	ctcagcactttggCATGtctt
IL8	interleukin 8	NM_000584	V$AP1	AP1.01	128	+	0.99	0.94	gatgaCTCAgg
					128	-	0.98	1.00	cctgaGTCAtc
				AP1.02	128	+	0.98	1.00	gaTGACtcagg

				AP1.03	128	+	0.98	1.00	gaTGACtcagg
				AP1FJ.01	128	+	0.97	1.00	gaTGACtcagg
			V$NFκB	CREL.01	83	-	0.93	1.00	agaggaaaTTCCacg
				CREL.01	83	+	0.97	1.00	cgtggaatTTCCtct
				HIVEP1.01	83	+	0.84	0.75	cgTGGAatttcctct
				NFKAPPAB.02	83	+	0.84	0.75	cgTGGAatttcctct
					83	+	0.92	0.82	cgTGGAatttcctct
				NFKAPPAB.03	83	-	0.87	0.78	agAGGAaattccacg
				NFKAPPAB65.01	83	+	0.98	1.00	cgtggaatTTCCtct
					83	-	0.88	1.00	agaggaaaTTCCacg
			V$STAT	STAT3.01	490	+	0.74	1.00	aacagTTCCtagaaactct
					490	-	0.79	0.75	agagtTTCTaggaactgtt
KCNN4	intermediate conductance Ca-activated K channel protein 1	NM_002250	V$AP1	AP1.01	68	+	0.98	1.00	tgtgaGTCAct
					68	-	0.97	0.94	agtgaCTCAca
				AP1.02	68	-	0.99	1.00	agTGACtcaca
				AP1.03	68	-	1.00	1.00	agTGACtcaca
				AP1FJ.01	68	-	0.99	1.00	agTGACtcaca
			V$NFκB	HIVEP1.01	471	-	0.91	1.00	taGGGAcatacctat
					-174	+	0.85	1.00	tgGGGAgcctccccc
					-174	-	0.83	1.00	ggGGGAggctcccca
				NFKAPPAB.01	471	-	0.92	1.00	taGGGAcatacctat
					-193	+	0.90	0.90	ctGGGGcctcccctg
				NFKAPPAB.02	471	-	0.83	1.00	taGGGAcatacctat
				NFKAPPAB.03	471	-	0.88	1.00	taGGGAcatacctat
					-174	+	0.88	1.00	tgGGGAgcctccccc
					-174	-	0.88	1.00	ggGGGAggctcccca
					-192	-	0.84	1.00	agGGGAggccccagg
				NFKAPPAB50.01	-193	-	0.91	1.00	caGGGGaggccccag
					-193	+	0.84	1.00	ctGGGGcctcccctg
			V$p53	p53.01	-392	+	0.70	1.00	ggcCATGggcggggatctggt
KRT18	keratin 18	NM_199187	V$NFκB	CREL.01	424	+	0.92	1.00	acaggccaTTCCacc
			V$p53	p53.02	164	+	0.92	1.00	tgctgtccgtgtcCATGcccg
KRT8	keratin 8	NM_002273	V$AP1	AP1FJ.01	-70	+	0.98	1.00	ggTGACccaga
			V$EGR	EGR1.02	170	-	0.88	1.00	tcaggtggGGGCagcag
					121	-	0.88	1.00	cccagaggGGGCtgggc
			V$NFκB	CREL.01	88	+	0.94	1.00	cgaggcctTTCCccc
					43	+	0.92	1.00	aaaggccaTTCCtga
				NFKAPPAB65.01	88	+	0.90	1.00	cgaggcctTTCCccc
			V$p53	p53.01	154	+	0.66	0.75	agtCCTGcccggaggtggcag
				p53.03	326	+	0.97	0.92	tgggcggcccaggCAAGtctg
MLPH	melanophilin	NM_024101	V$EGR	EGR1.01	159	-	0.84	0.83	cagtgcgggGGAGagcc
					137	+	0.80	1.00	tcccgcgcgGGCGcccc
			V$NFκB	NFKAPPAB50.01	163	+	0.83	1.00	ccGGGGctctccccc
Pfs2	DNA replication complex GINS protein PSF2	NM_016095	V$EGR	EGR1.01	228	-	0.82	1.00	ccgcccgtgGGCGtgca
				EGR1.02	220	+	0.92	1.00	cacgggcgGGGCggggc
					203	+	0.97	1.00	cggggtggGGGCggggc
					180	+	0.92	1.00	gcgggccgGGGCggggc
					110	-	0.95	1.00	gggcggggGGGCggaga
					37	+	0.91	1.00	tccggccgGGGCgggct
			V$p53	p53.02	200	+	0.92	0.82	ggtgggggcggggCCTGtccg
PLK1	polo-like kinase 1 (Drosophila)	NM_005030	V$EGR	EGR1.02	320	+	0.87	0.84	cctggaggAGGCgcaag
			V$NFκB	CREL.01	480	-	0.92	1.00	tcaggtgaTTCCtac

			V$STAT	STAT3.01	293	+	0.77	1.00	gagctTTCCcggacgcccg
					293	-	0.74	0.75	cgggcGTCCgggaaagctc
PORIMIN	pro-oncosis receptor inducing membrane injury gene	NM_052932	V$EGR	EGR1.01	63	+	0.86	0.83	gcctgcgagGGAGgccg
					17	-	0.84	1.00	tggcgcgggGGCGggct
				EGR1.02	17	-	0.92	1.00	tggcgcggGGGCgggct
					-1	-	0.90	1.00	cgcagccgGGGCggcgg
					-9	-	0.88	0.79	agccggggCGGCggggc
					-21	-	0.94	1.00	cctggaggGGGCgtcgc
			V$NFκB	NFKAPPAB65.01	83	+	0.88	0.83	tctggggtTCCCaag
PPP1R12A	protein phosphatase 1, regulatory (inhibitor) subunit 12A	NM_002480	V$EGR	EGR1.01	367	-	0.82	0.83	aactgcggcGGTGgtgg
					42	-	0.79	0.80	tatcgcgagGGGGggaa
				EGR1.02	416	-	0.89	1.00	ggcagcggGGGCtggga
					231	+	0.89	1.00	cgtgctggGGGCgggag
					56	-	0.89	0.84	gaagggggAGGCggaga
			V$NFκB	NFKAPPAB.01	477	-	0.88	0.90	ctGGGGgctcccggg
				NFKAPPAB50.01	477	-	0.84	1.00	ctGGGGgctcccggg
PTPRJ	protein tyrosine phosphatase, receptor type, J	NM_002843	V$EGR	EGR1.02	212	+	0.87	1.00	gggcggagGGGCgggcg
					203	+	0.87	0.84	ggcgggcgAGGCgggag
					150	+	0.98	1.00	ctcggaggGGGCggggg
					144	+	0.91	1.00	gggggcggGGGCagcgg
					128	+	0.88	0.79	gcgggcggCGGCgaggg
					87	+	0.93	1.00	gccggccgGGGCgggag
					41	+	0.87	1.00	aagggaggGGGCcgagc
					-16	+	0.87	0.84	cgcggaggAGGCagcgg
			V$NFκB	NFKAPPAB50.01	168	+	0.86	1.00	gaGGGGatccgcggc
RAB17	RAB17, member RAS oncogene family	NM_022449	V$EGR	EGR1.02	380	+	0.90	0.79	cactgtggTGGCggagc
					62	-	0.96	1.00	cggggtggGGGCgtggc
			V$NFκB	HIVEP1.01	87	-	0.87	1.00	atGGGAgactccagg
				NFKAPPAB.03	87	-	0.85	1.00	atGGGAgactccagg
RAD54L	RAD54-like (S. cerevisiae)	NM_003579	V$AP1	AP1.03	183	-	0.96	1.00	cgTGACttaga
			V$EGR	EGR1.01	264	-	0.82	0.83	ggtggcgggGGAGgggc
					255	-	0.80	0.83	gggggcgggGGTGgcgg
					249	-	0.85	1.00	cggggcgggGGCGgggg
					197	-	0.81	1.00	agatgggagGGCGgggc
				EGR1.02	434	-	0.90	1.00	gaggggagGGGCggaat
					344	-	0.91	1.00	gcggggagGGGCggggc
					334	-	0.89	1.00	gctggacgGGGCgggga
					315	-	0.89	1.00	gcggggcgGGGCgagaa
					269	-	0.91	1.00	cgggggagGGGCggggc
					258	-	0.91	0.79	ggcgggggTGGCggggg
					249	-	0.98	1.00	cggggcggGGGCggggg
					243	-	0.90	1.00	gaggggcgGGGCggggg
RPN2	ribophorin II	NM_002951	V$AP1	AP1.02	-149	-	0.96	1.00	gcTGACttccg
			V$EGR	EGR1.02	421	-	0.86	0.84	gacctgggAGGCggagg
			V$NFκB	NFKAPPAB.01	21	-	0.89	1.00	ccGGGAgctaccggg
			V$STAT	STAT3.01	18	+	0.78	0.75	ggtagCTCCcggaatagga
					18	-	0.75	1.00	tcctaTTCCgggagctacc
SHANK2	SH3 and multiple ankyrin repeat domains 2	NM_133266	V$EGR	EGR1.01	313	-	0.81	1.00	agcagcggcGGCGtcgg
					286	-	0.79	1.00	aacggcggcGGCGgcag
				EGR1.02	286	-	0.88	0.79	aacggcggCGGCggcag
					192	-	0.86	1.00	acatggtgGGGCggctg
					145	-	0.88	1.00	cagggtggGGGCaggca

			V$NFκB	NFKAPPAB.03	-4	-	0.85	1.00	cgGGGAcgttcatca
				NFKAPPAB65.01	367	+	0.88	0.83	cccgggctTCCCtgg
			V$STAT	STAT3.01	425	-	0.76	1.00	tgcttTTCCttgaaggggg
					-63	+	0.75	0.75	cgctgTCCCcggaattctc
					-63	-	0.78	1.00	gagaaTTCCggggacagcg
SNCG	synuclein, γ (breast cancer-specific protein 1)	NM_003087	V$EGR	EGR1.02	145	-	0.95	1.00	tgcagtggGGGCgggga
SRPX2	sushi-repeat-containing protein, X-linked 2	NM_014467	V$AP1	AP1.01	140	+	0.98	1.00	gctgaGTCAta
					140	-	0.99	0.94	tatgaCTCAgc
				AP1.02	73	-	0.98	1.00	gaTGACtaaca
				AP1.03	140	-	0.95	1.00	taTGACtcagc
					73	-	0.98	1.00	gaTGACtaaca
STC1	stanniocalcin 1	NM_003155	V$p53	p53.01	453	-	0.66	0.75	gcaCATTctcggttctgtttt
			V$STAT	STAT3.01	351	+	0.79	1.00	tccatTTCCccaagtggc
					241	+	0.74	1.00	cttacTTCCacgaaaatag
					241	-	0.77	0.75	ctattTTCGtggaagtaag
					159	+	0.78	0.75	ttaatTTGCtggaaaatat
					159	-	0.79	1.00	atattTTCCagcaaattaa
STK6	serine/threonine kinase 6	NM_198437	V$STAT	STAT3.01	391	+	0.75	0.75	gctctCTCCtggaaaaatg
TOMM34	translocase of outer mitochondrial membrane 34	NM_006809	V$EGR	EGR1.01	-53	-	0.80	1.00	gcaagcgccGGCGgcga
					-11	-	0.95	1.00	cggcgaggGGGCggggc
			V$NFκB	HIVEP1.01	20	+	0.85	1.00	gcGGGAcgctccggg
					-71	+	0.84	0.75	atGAGAgtttccgca
				NFKAPPAB.01	-53	-	0.88	0.82	ggGGGCcatcccgtg
				NFKAPPAB.02	20	+	0.84	1.00	gcGGGAcgctccggg
					-71	+	0.83	0.75	atGAGAgtttccgca
				NFKAPPAB.03	20	+	0.87	1.00	gcGGGAcgctccggg
TXNRD1	thioredoxin reductase 1	NM_182743	V$AP1	AP1.02	429	+	0.98	1.00	acTGACtaatc
				AP1.03	495	-	0.96	1.00	ccTGACtaatc
					429	+	0.98	1.00	acTGACtaatc
			V$EGR	EGR1.02	132	+	0.90	1.00	taggttggGGGCggcta
			V$NFκB	CREL.01	443	-	0.92	1.00	tttggcagTTCCcta
				HIVEP1.01	443	+	0.87	1.00	taGGGAactgccaaa
				NFKAPPAB.01	443	+	0.91	1.00	taGGGAactgccaaa
				NFKAPPAB.03	443	+	0.87	1.00	taGGGAactgccaaa
YAP1	Yes-associated protein 1, 65kDa	NM_006106	V$EGR	EGR1.01	398	+	0.81	1.00	ctgagcgcgGGCGaagg
					212	+	0.84	1.00	ggcggcgcgGGCGggcg
					-57	+	0.85	1.00	cagggcgggGGCGgagg
					-224	+	0.80	0.80	gtgcgcgtcGGGGgagg
					-300	-	0.87	1.00	ggctgcgaaGGCGgctg
				EGR1.02	266	+	0.87	1.00	cgcggtgcGGGCgggcg
					232	+	0.88	0.84	ccgagaggAGGCggggc
					218	+	0.87	0.79	ggccgcggCGGCgcggg
					212	+	0.87	1.00	ggcggcgcGGGCgggcg
					-57	+	0.97	1.00	cagggcggGGGCggagg
					-63	+	0.89	0.84	gggggcggAGGCgccgg
					-71	+	0.88	1.00	aggcgccgGGGCggggg
					-309	-	0.91	1.00	ccctgcggGGGCtgcga
			V$NFκB	CREL.01	242	-	0.91	1.00	ctcggctcTTCCttc
ZNF239	zinc finger protein 239	NM_005674	V$STAT	STAT3.01	128	+	0.75	1.00	ttattTTCCcagtaatgtt

Shown are binding sites of five transcription factors (TF), p53, NF-κB, AP-1, STAT3 and EGR1, predicted by using Genomatix Suite 3.4.1.

[1] refers to transcription factor family defined by Genomatix Suite 3.4.1. V$ indicates TF family from vertebrates.

[2] refers to transcription factor binding matrices within the TF family from Genomatix Suite 3.4.1

[3] refers to distance (bp) to transcriptional start site; positive is for upstream; negative is for downstream.

[4] The matrix or core similarity was calculated for whole motif or core sequence (capital letters) [112].
The similarity of 1.0 is only reached when the highest conserved bases of a matrix match exactly in the sequence.
